# DL‐3‐n‐Butylphthalide Protects Mitochondria Against Ischemia/Hypoxia Damage via Suppressing GCN5L1‐Mediated Drp1 Acetylation in Neurons and Mouse Brains

**DOI:** 10.1002/cns.70682

**Published:** 2025-11-30

**Authors:** Haitao Zhang, Ning Zhang, Xiaotong Yang, Jiejie Zhang, Xiaoli Ge, Lei Wang, Shan Wang, Ya Wen

**Affiliations:** ^1^ Department of Neurology The Second Hospital of Hebei Medical University Shijiazhuang Hebei China; ^2^ Key Laboratory of Clinical Neurology, Ministry of Education Hebei Medical University Shijiazhuang Hebei China; ^3^ Neurological Laboratory of Hebei Province Shijiazhuang Hebei China; ^4^ Department of Human Anatomy Institute of Medicine and Health, Hebei Medical University Shijiazhuang Hebei China

**Keywords:** DL‐3‐n‐butylphthalide, Drp1, GCN5L1, ischemic stroke, mitochondrial dysfunction

## Abstract

**Background:**

Mitochondrial dysfunction is an initial event of the cascade reactions triggered by ischemic stroke, contributing to the pathogenesis of ischemic brain injury. DL‐3‐n‐butylphthalide (NBP), a compound originally isolated from the seeds of *Apium graveolens* Linn, exerts neuroprotective effects by improving mitochondrial function in ischemic brain tissues; however, the exact molecular mechanisms underlying its action remain poorly understood.

**Methods:**

The OGD‐exposed neuronal cells and dMCAO mice were used to investigate the effects of ischemia/hypoxia on mitochondrial function and the protective action of NBP on mitochondrial damage. Co‐immunoprecipitation and immunofluorescence staining were performed to identify the interaction between Drp1 and GCN5L1. Western blotting, immunofluorescence and immunohistochemical staining were conducted to detect the expression of GCN5L1, Drp1, ERK1/2, Bax, Bcl2, and caspase‐3. The mitochondrial function was analyzed by measuring mitochondrial ROS, ATP production, mitochondrial membrane potential (MMP) and mPTP opening.

**Results:**

We observed that mitochondrial dysfunction occurs in OGD‐treated neuronal cells and brain tissues of dMCAO mice, as evidenced by the alteration in the mPTP, MMP, ATP content, and ROS levels, which are accompanied by a significant increase in mitochondrial fission and neuronal apoptosis, as shown by TUNEL staining and the changes in Bcl‐2, Bax and caspase‐3 expression. Importantly, NBP intervention significantly attenuates ischemia/hypoxia‐induced mitochondrial dysfunction and cellular apoptosis in the neuron and mouse brains. Mechanistically, NBP not only reverses the upregulation of Drp1 and GCN5L1 expression by ischemia/hypoxia, but also inhibits the ischemia/hypoxia‐induced phosphorylation of Drp1 by blocking the ERK1/2 signaling, which in turn suppresses the interaction between Drp1 and GCN5L1, thereby decreasing Drp1 acetylation by GCN5L1 and excessive mitochondrial fission.

**Conclusion:**

Our findings provide a novel insight into the molecular mechanism whereby NBP protects mitochondria against ischemia/hypoxia damage, offering a promising drug for mitochondria‐targeting therapeutics for ischemic stroke.

## Graphical abstract

This study provides a novel insight into the molecular mechanism whereby NBP protects mitochondria against ischemia/hypoxia damage, offering a promising drug for mitochondria‐targeting therapeutics for ischemic stroke.

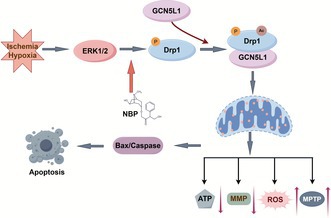

## 1 Introduction

Ischemic stroke is a severe and life‐threatening disease, and is one of the common causes of mortality and disability worldwide [1]. Due to their high energy consumption, neuronal cells require a constant supply of glucose and oxygen. When the cerebral blood supply is disrupted due to an occlusion of cerebral arteries, neuronal cells are particularly vulnerable to ischemic and hypoxic injury [2, 3]. Within the few hours of stroke onset, glucose and oxygen in brain tissues are deprived, leading to a complex series of pathophysiological processes including oxidative stress, inflammation, excitotoxicity, and apoptotic cell death [4]. Mitochondrial oxidative phosphorylation that generates ATP is essential for the maintenance of normal neuron functions and continuous cell survival. Following ischemic stroke, ATP synthesis is disturbed owing to the depletion of brain tissue oxygen, contributing to mitochondrial dysfunctions including mitochondrial depolarization that initiates excessive ROS production, the mitochondrial permeability transition pore (mPTP) opening, and release of cytochrome C from the mitochondrion into the cytosol [5]. Accumulating evidence has indicated that mitochondrial dysfunction plays a central role in the pathological process of ischemic stroke.

Mitochondrial dysfunction is tightly linked to structural and morphological alterations of mitochondria, which are dynamically regulated by the coordination of fission and fusion; that is, mitochondrial dynamics [6, 7]. It is well known that mitochondrial dynamics are regulated by a family of GTPases, in which mitofusin 1/2 (Mfn1/2) and optic atrophy 1 (Opa1) regulate mitochondrial fusion, whereas dynamin‐related protein 1 (Drp1) plays a crucial role in the modulation of mitochondrial fission [8, 9]. When ischemic stroke occurs, mitochondrial impairment leads to an imbalance in mitochondrial dynamics, resulting in excessive fission of mitochondria. The fission of the mitochondria is considered an initial event in apoptotic cell death because mitochondrial oxidative phosphorylation is the first one ceased due to the depletion of oxygen [10, 11]. These studies suggest that Drp1‐mediated mitochondrial fission exerts an indispensable role in cerebral ischemia/hypoxia injury. Previous works have shown that Drp1 activity is modulated by several post‐translational modifications such as phosphorylation, ubiquitination, SUMOylation, S‐nitrosylation, and others [12, 13]. Moreover, the serine 616 (Ser616) site of Drp1 is known to be phosphorylated by cyclin‐dependent kinase 1 (Cdk1), calmodulin‐dependent protein kinase II (CaMKII), and ERK in cardiomyocytes, fibroblasts, and colorectal cancer, and this site phosphorylation results in excessive mitochondrial fission and cell apoptosis [12]. Our recent research suggests that neuronal and cerebral ischemia/hypoxia promote GCN5L1 interaction with Drp1 in an AMPK‐mediated Drp1 phosphorylation‐dependent manner, and thus enhance Drp1 acetylation, leading to excessive mitochondrial fission [14]. Despite considerable advances in understanding the regulation of mitochondrial dynamics, a cascade of intricate reactions triggered by neuronal and cerebral ischemia/hypoxia has not yet been fully understood.

Mitochondrial dysfunction contributes to the pathogenesis of cerebral ischemia, causing neuronal impairment and cell death following ischemic stroke. Therefore, mitochondria‐targeted therapies may represent promising therapeutic strategies for ischemic stroke. Besides intercellular mitochondria transfer [15], various pharmacological agents, such as nicotinamide mononucleotide (NMN) [16], Metformin [17], and miRNAs [18], have been developed to improve mitochondrial function. Although numerous efforts have been made to reduce ischemia‐induced mitochondrial injury, the number of patients living with the consequences of ischemic stroke is steadily increasing. Mitochondrial targeting by pharmacological agents is still challenging. DL‐3‐n‐butylphthalide (NBP) is a chiral compound synthesized from L‐3‐n‐butylphthalide, which was originally extracted from the seeds of *Apium graveolens* Linn [19]. NBP was approved by the State Food and Drug Administration of China (SFDA) in 2002 for the treatment of acute ischemic stroke. Clinical studies have shown that NBP can effectively improve central nervous system function after ischemic stroke and promote the recovery of impaired neurological function of patients with acute cerebral ischemia [20]. Accumulating evidence suggests that NBP has a wide range of pharmacological properties and targets, and that its neuroprotective function involves multiple mechanisms, such as promoting cerebral blood flow, improving mitochondrial function, reducing oxidative stress damage, inhibiting inflammatory responses, and suppressing neuronal apoptosis [21, 22]. Studies have shown that NBP and NAD^+^ have similar neuroprotective and antioxidant effects on cerebral ischemia, and that the protective effect of NBP is achieved through SIRT1 [20]. In addition, NBP can promote the ubiquitination and degradation of hypoxia‐inducible factor 1α (HIF‐1α), the key transcription factor that regulates the Bax gene, thereby inhibiting cell apoptosis and facilitating neurological function recovery following cerebral ischemia [23]. Studies also found that NBP may act directly on mitochondrial complex IV to increase its activity [22]. Although recent research has identified NBP as an effective agent to improve mitochondrial dysfunction caused by ischemic stroke, the exact molecular mechanisms underlying its action remain largely unknown.

In this study, the specific mechanisms by which NBP regulates mitochondrial function were investigated in hypoxic neuronal cells and ischemic mouse brain tissues. The novel mechanisms underlying the protective effect of NBP against ischemia/hypoxia‐induced mitochondrial damage were elucidated using in vivo and in vitro experiments, providing a promising drug for mitochondria‐targeting therapeutics for ischemic stroke.

## 2 Materials and Methods

### 2.1 Cell Culture, OGD and Drug Treatment

Neuro‐2a cells were obtained from the American Type Culture Collection (ATCC) and were cultured in Dulbecco's Modified Eagle's Medium (DMEM) (Gibco) supplemented with 10% fetal bovine serum (FBS) (BIOEXPLORER Life Sciences, BS1612‐105) and antibiotics (100 μg/mL streptomycin and 100 U/mL penicillin) (Gibco). Cells were cultured at 37°C in a 5% CO_2_ atmosphere. To induce oxygen–glucose deprivation (OGD), the cells were exposed to a hypoxic environment (94% N_2_, 1% O_2_, 5% CO_2_) in glucose‐free DMEM (Gibco) to mimic an in vitro model of permanent cerebral ischemia. NBP (purity 98.8%) was obtained from Shijiazhuang Pharmaceutical Co. Ltd., China, and was dissolved in DMSO. The cells were treated with different doses of NBP for varying durations according to the experimental requirements. For the positive control drugs, 0.5 mM nicotinamide mononucleotide (NMN) (Sigma‐Aldrich, St. Louis, MO, USA) or 100 μM resveratrol (Sigma‐Aldrich, St. Louis, MO, USA) was added to the OGD‐exposed Neuro‐2a cell culture medium and incubated for 48 h. Then the cells were collected and used in the subsequent experiments.

### 2.2 Animal Study

Male C57BL/6J mice, aged 6–8 weeks and weighing 20–25 g, were provided by the Department of Laboratory Animal Science, Hebei Medical University (SCXK[Ji] 2022‐001) and were housed under specific pathogen‐free conditions. All experimental procedures were performed in strict accordance with the guide for the Care and Use of Laboratory Animals (NIH). A permanent distal middle cerebral artery occlusion (dMCAO) model was established: Mice were anesthetized under 3.5% isoflurane and maintained on 1.5% isoflurane throughout the surgical procedure. Under sterile conditions, a midline incision was made to expose and isolate the right common carotid artery (CCA), external carotid artery (ECA), and internal carotid artery (ICA) using a surgical microscope. The CCA and ECA were ligated, a filament was gently introduced into the lumen of the CCA, then delivered to the middle cerebral artery (MCA) and secured. For sham‐operated control mice, the same procedure was followed, except for the insertion of the filament. NBP (purity 98.8%) was obtained from Shijiazhuang Pharmaceutical Co. Ltd., China. As previously reported, NBP was dissolved in corn oil (Aladdin, C116023), and administered to dMCAO mice at a dose of 60 mg/kg/day via oral gavage, and mice were subjected to continuous administration for 3 days [24, 25]. The cerebral cortex of dMCAO mice was collected for immunohistochemistry (IHC) and immunofluorescence (IF) staining, as well as for protein extraction and immunoblotting.

### 2.3 TTC Staining of Brain Sections

The fresh brains from sham‐operated, dMCAO, and NPB‐treated dMCAO mice were sliced into coronal sections with 2‐mm thickness, incubated in a 2% solution of 2, 3, 5‐triphenyltetrazolium chloride (TTC) (Sigma‐Aldrich, St Louis, MO, USA) at 37°C for 20 min, followed by fixation with 4% paraformaldehyde. TTC‐stained sections were photographed and the images were analyzed using Image‐Pro Plus 6.0 (Media Cybernetics, MD, USA), and the infarct volumes were calculated as follows: Corrected Infarct Volume (%) = [(Contralateral Hemisphere Area − Ipsilateral Non‐Infarcted Area)/Contralateral Hemisphere Area] × 100.

### 2.4 Neurological Deficit Scores

For the Longa neurological scores, a 5‐point scale is employed to assess neurological deficits. A score of 0 indicates no neurological deficit. A score of 1 indicates a mild focal neurological deficit, manifested by incomplete extension of the paralyzed forepaw. A score of 2 indicates a moderate focal neurological deficit, manifested by circling to the paralyzed side. A score of 3 indicates severe focal functional impairment, with the mouse falling to the paralyzed side. A score of 4 indicates the mouse's inability to walk spontaneously, exhibiting a decreased level of consciousness.

### 2.5 Determination of Cell Viability

The CCK‐8 assay was used to evaluate the viability of Neuro‐2a cells under different concentrations of NBP. Briefly, Neuro‐2a cells were seeded at a density of 5000 cells/well in a 96‐well plate and incubated for 24 h. The culture medium was then replaced with DMEM containing 10% FBS and different concentrations of NBP. After incubation for various time periods, 10 μL of CCK‐8 reagent (Beyotime, C0048M) was added to each well. The absorbance was measured at 450 nm to record cell viability.

### 2.6 JC‐1 Assay

The mitochondrial membrane potential was assessed using the Enhanced Mitochondrial Membrane Potential Assay Kit with JC‐1 (Beyotime, C2003S). Neuro‐2a cells were incubated with the JC‐1 fluorescent probe at 37°C for 20 min. Fluorescence intensity was detected by confocal microscopy, and changes in mitochondrial membrane potential were evaluated based on the transition in fluorescence emission.

### 2.7 mPTP Opening Assay

The mitochondrial permeability transition pore (mPTP) opening was assessed using the Mitochondrial Permeability Transition Pore Assay Kit (Beyotime, C2009S). Neuro‐2a cells were incubated with the membrane‐permeant fluorescent probe Calcein AM and the fluorescence quencher CoCl_2_ at 37°C for 30 min. Fluorescence intensity was measured by confocal microscopy, and the extent of mPTP opening was determined based on the decrease in Calcein AM fluorescence, which indicated increased mitochondrial membrane permeability.

### 2.8 DHE Fluorescence Assay

The level of reactive oxygen species (ROS) was determined using Dihydroethidium (DHE) (Beyotime, S0063). Neuro‐2a cells were incubated with the fluorescent probe DHE for 30 min at 37°C in the dark. Fluorescence intensity was detected using a fluorescence microscope (OLYMPUS, BX53F2) to assess ROS levels. For brain tissue analysis, sections (4 μm thick) from each group were prepared and incubated with DHE at 37°C in the dark for 30 min. The tissue sections were then observed under a fluorescence microscope to evaluate ROS production.

### 2.9 Detection of ATP

ATP levels were measured using the ATP Assay Kit (Beyotime, S0026). Cells were seeded in 6‐well plates, and 200 μL of lysis buffer was added to each well. For tissue samples, 200 μL of lysis buffer was added per 20 mg of tissue. After lysis, the samples were centrifuged at 12,000 *g* for 5 min at 4°C, and the supernatant was collected. The supernatant was mixed with the ATP detection working solution, and the luminescence intensity was measured using a microplate reader. ATP content was calculated based on a standard curve. Protein concentration was determined using the BCA Protein Assay Kit (SEVEN, SW101‐02). The relative ATP content was expressed as ATP levels normalized to protein concentration (nmoL ATP/mg protein).

### 2.10 Western Blotting

Protein extraction from cells and tissues was performed using cold lysis buffer containing PMSF and RIPA at a ratio of 1:100. The lysates were centrifuged, and the protein supernatant was collected. Protein concentration was determined using BCA. Proteins were separated by SDS‐PAGE and transferred onto polyvinylidene fluoride (PVDF) membranes (Millipore). The membranes were blocked with 5% non‐fat milk at room temperature for 2 h and then incubated overnight at 4°C with the following primary antibodies: rabbit anti Drp1 (1:1000) (Abcam, ab9787), Bcl‐2 (1:500) (Proteintech, 26,593–1‐AP), ERK1/2 (1:1000) (Proteintech, 66,470–2‐Ig), p‐ERK1/2 (1:1000), p‐Drp1 (1:1000) (Cell Signaling Technology, 4494), GCN5L1 (1:500) (Proteintech, 19,687–1‐AP), mouse anti Bax (1:2000) (Proteintech, 6027–1‐Ig), Caspase‐3 (1:1000) (Proteintech, 66,470–2‐Ig), and β‐actin (1:10000) (Proteintech, 66,009–1‐Ig). After washing, the membranes were incubated with appropriate secondary antibodies at room temperature for 90 min. Protein bands were visualized using an ECL detection system. The density of Western blot bands was measured using Image J software and normalized to β‐actin.

### 2.11 Immunohistochemical Staining

Brain tissues were immediately fixed in 4% paraformaldehyde for 48 h after collection, followed by dehydration through an ethanol gradient and embedding in paraffin. According to the immunohistochemistry kit instructions (Zhongshan Golden Bridge Biotechnology, Beijing, China), brain sections or fixed cells underwent antigen retrieval. Endogenous peroxidase activity was blocked by incubation with a peroxidase blocker. The sections were then incubated overnight at 4°C with the following primary antibodies: rabbit anti‐Drp1 (1:700, Abcam), GCN5L1 (1:300, Proteintech), Bcl‐2 (1:50, Proteintech), p‐ERK1/2 (1:200, Proteintech), mouse anti‐Bax (1:1000, Proteintech), and Caspase‐3 (1:100, Proteintech). After washing with PBS, the sections were incubated with an enhancer solution and secondary antibody at 37°C for 20 min, followed by DAB staining for visualization. Immunohistochemistry staining was quantified by H‐score.

### 2.12 TUNEL Staining

The sections were dewaxed and rehydrated after formalin fixation and paraffin embedding. The slides were incubated with Proteinase K (20 μg/mL) at room temperature for 20 min, followed by three washes with PBS. TUNEL detection solution was prepared by mixing TdT enzyme, fluorescent dye, and detection liquid (Vazyme, A112). Samples were incubated with this solution under 37°C in the dark for 60 min, followed by PBS washes. Finally, slides were stained with DAPI and analyzed under a fluorescence microscope.

### 2.13 Immunofluorescence Staining

After appropriate treatment, Neuro‐2a cells were fixed with 4% paraformaldehyde for 15 min and permeabilized with 0.5% Triton X‐100 for 5 min, followed by three washes with PBS. After blocking with goat serum for 30 min, the cells were incubated overnight at 4°C with primary antibodies against Drp1 (1:200, Abcam, ab9787), GCN5L1 (1:50, Proteintech), Bax (1:200, Proteintech), Caspase‐3 (1:50, Proteintech), Bcl‐2 (1:50, Proteintech), and p‐ERK1/2 (1:50, Proteintech). Subsequently, the cells were washed and subjected to secondary permeabilization with 0.5% Triton X‐100, followed by staining with 4′,6‐diamidino‐2‐phenylindole (DAPI) (SouthernBiotech, 0100–20). Images were captured using a confocal microscope. Immunofluorescence signal intensity was quantified by Image J software (USA).

### 2.14 Cell Apoptosis

Flow cytometry was employed to detect cell apoptosis using Annexin V/PI staining (Beyotime, C1062M). Briefly, cells were seeded in a 6‐well plate. After treatment, the cells were collected and washed twice with PBS. Then, the cells were incubated with Annexin V‐FITC and PI in the dark at room temperature for 30 min, then the data analysis was performed on the flow cytometer (Agilent).

### 2.15 Co‐Immunoprecipitation (Co‐IP)

Protein A/G magnetic beads (MCE, HY‐K0202) were washed three times, and then incubated with the target antibody in buffer at room temperature for 30 min. After incubation, the beads were washed four times and combined with 300 μg of cellular or tissue protein, followed by another 30‐min incubation at room temperature. After washing, cell lysate supernatants were combined with the beads to form an immunomagnetic beads‐antibody–antigen complex. Following additional washes, the complex was resuspended in loading buffer for subsequent western blot analysis of endogenous protein interactions.

### 2.16 Statistical Analysis

Statistical analysis was conducted using GraphPad Prism9 software. Data are expressed as means ± standard deviation. One‐way ANOVA was performed, followed by Tukey's multiple comparisons test. Values of *p* < 0.05 were considered statistically significant.

## 3 Results

### 3.1 Mitochondrial Dysfunction Occurs in Ischemia/Hypoxia‐Induced Injury in Neuronal Cells and Brain Tissues

**FIGURE 1:**
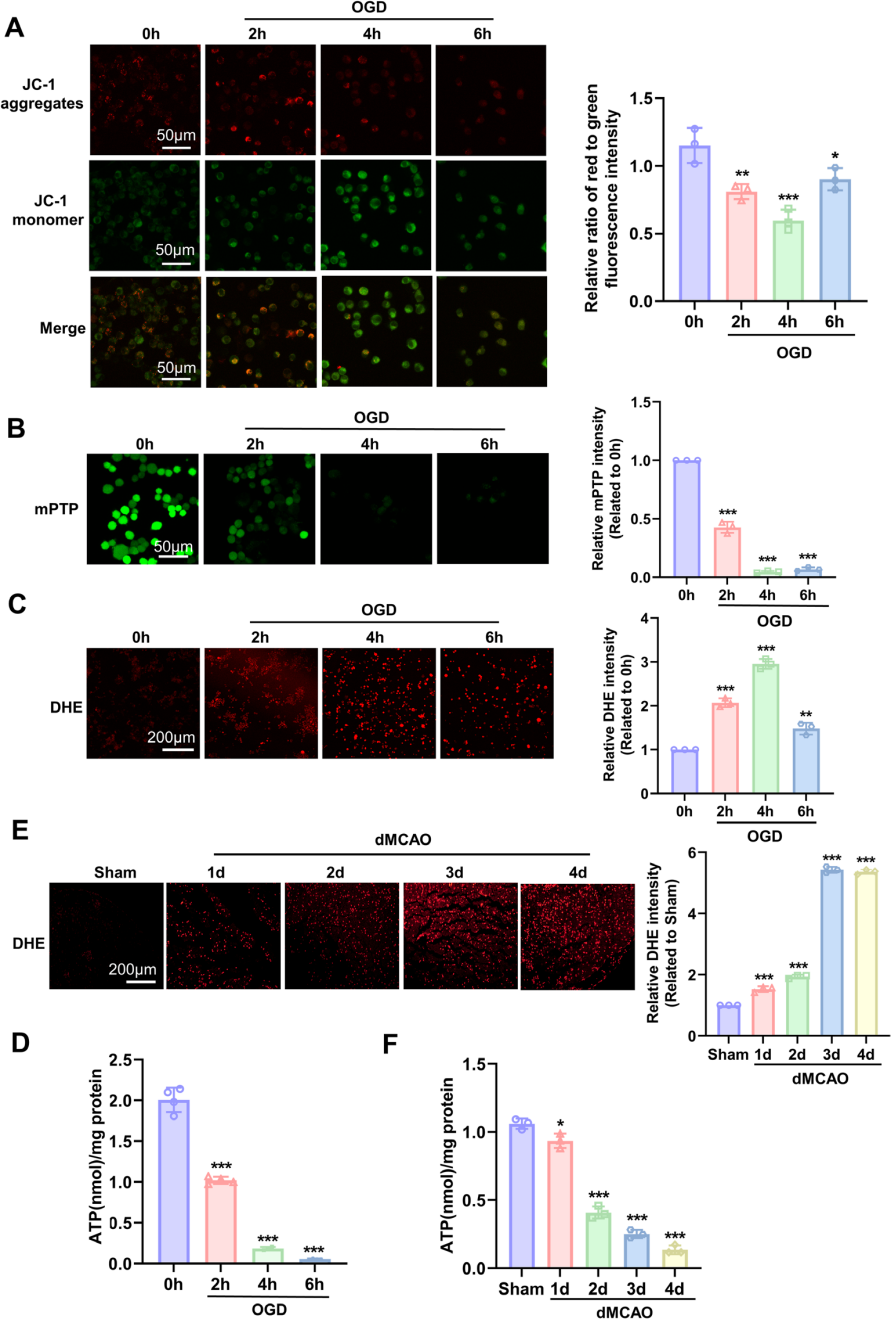
Ischemia/hypoxia resulted in mitochondrial dysfunction in neuronal cells and mouse brain tissue. (A) Neuro‐2a cells were exposed to OGD for different times, and then nitochondrial membrane potential (MMP) was detected by MMP Assay Kit with JC‐1. Relative ratio of red to green JC‐1 fluorescence intensity was measured to reflect MMP. The representative fluorescence images are shown on the left. Scale bars = 50 μm. A quantitative analysis of fluorescence intensity measured by Image J software is shown on the right. Data represent the mean ± SD, *p < 0.05, **p < 0.01, and ***p < 0.001 versus 0 h. *n* = 3. (B) Neuro‐2a cells were treated as in (A), and mitochondrial permeability transition pore (mPTP) opening was assessed by the quenching of calcein fluorescence with cobalt. The representative fluorescence images are shown on the left. Scale bar = 50 μm. The right panel shows quantitative analysis of fluorescence intensity. ***p < 0.001 versus 0 h. *n* = 3. (C) Neuro‐2a cells were treated as in (A), and ROS levels were measured by dihydroethidium (DHE) fluorescent probe. The representative fluorescence images are shown on the left. Scale bar = 200 μm. The right panel shows quantitative analysis of fluorescence intensity. **p < 0.01, ***p < 0.001 versus 0 h. *n* = 3. (D) Neuro‐2a cells were treated as in (A), and the ATP content was determined by using an ATP assay kit. Data are represented as mean ± SD, ***p < 0.001 versus 0 h. *n* = 3 for each group. (E) Mice were subjected to sham or dMCAO surgery, and the ischemic area of the cerebral cortex was collected and sectioned after 1, 2, 3, and 4 days. ROS levels were measured by dihydroethidium (DHE) fluorescent probe. The representative fluorescence images are shown on the left. Scale bar = 200 μm. The right panel shows quantitative analysis of fluorescence intensity. ***p < 0.001 versus Sham. *n* = 3. (F) The cerebral cortex tissues on the ischemic side of mice subjected to dMCAO for different times were homogenized, and the ATP content was measured as described in (D). Data are represented as mean ± SD, *p < 0.05, ***p < 0.001 versus Sham. *n* = 3.

Mitochondrial dysfunction is the most important mechanism of cerebral damage caused by ischemic stroke [5]. To corroborate the occurrence of mitochondrial dysfunction in ischemic neuron and brain tissues, we first established the in vitro and in vivo models of cerebral ischemic injury via oxygen–glucose deprivation (OGD) of cultured Neuro‐2a cells as well as via distal middle cerebral artery occlusion (dMCAO) produced by transcranial electrocoagulation in mice. Mitochondrial function was evaluated by analyzing mitochondrial membrane potential (MMP), the mitochondrial permeability transition pore (mPTP), reactive oxygen species (ROS), and ATP content in OGD‐treated Neuro‐2a cells and in the ischemic brain tissues induced by dMCAO. The results showed that MMP, detected by using the fluorescent dye JC‐1, was significantly decreased in Neuro‐2a cells subjected to OGD treatment for 4 h, as shown in Figure 1A, where red and green fluorescence denotes high and low MMP, respectively. Simultaneously, OGD‐induced mPTP opening was accompanied, as assessed by co‐loading with Calcein AM and CoCl2 (Figure 1B). Further, we investigated whether alterations in MMP and mPTP affect ROS production. We employed dihydroethidium (DHE) staining to measure total superoxide/ROS levels and found that ROS production increased with prolonged OGD treatment and reached a peak at 4 h (Figure 1C). Mitochondria are the primary organelles for the generation of ATP. Next, we examined the impact of ischemia/hypoxia on ATP production in OGD‐treated Neuro‐2a cells and confirmed that cellular ATP content was time‐dependently attenuated over the observed duration (Figure 1D). To further validate the findings of the in vitro experiments, we assessed ROS levels in the ischemic brain tissues induced by dMCAO in mice by using DHE staining. As expected, ROS generation was dramatically increased after 3 days of dMCAO (Figure 1E), with a concomitant decrease of ATP content in ischemic brain tissues of dMCAO mice with prolonged ischemic time (Figure 1F). Together, these results suggest that OGD‐ and dMCAO‐induced ischemia/hypoxia injury leads to mitochondrial dysfunction in the neuronal cells and brain tissues.

### 3.2 Mitochondrial Dysfunction Elicited by Ischemia/Hypoxia Contributes to Neuronal Cell Apoptosis

**FIGURE 2:**
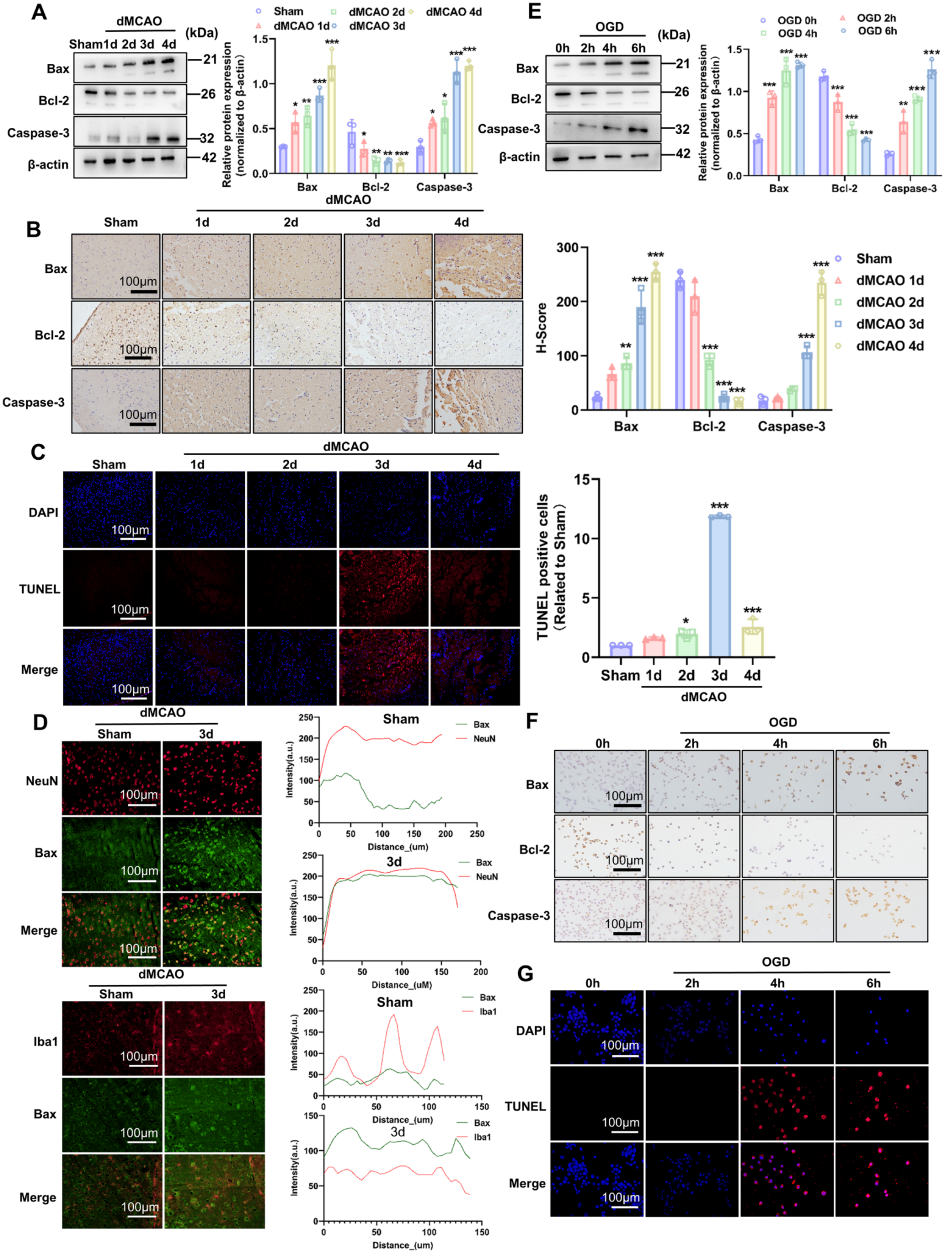
Mitochondrial dysfunction contributed to neuronal cell apoptosis. (A) Protein extracts of mouse brain subjected to dMCAO for different times were collected and analyzed by Western blot for Bax, Bcl‐2, and Caspase‐3. β‐Actin was included as a loading control. A representative blot is shown on the left, whereas band intensities that were measured and normalized to β‐Actin are shown on the right. Data are represented as mean ± SD, *p < 0.05, **p < 0.01, and ***p < 0.001 versus Sham. *n* = 3. (B) Immunohistochemical staining of Bax, Bcl‐2 and Caspase‐3 on brain sections of mice subjected to dMCAO for different times. The representative images are shown on the left. Scale bars = 100 μm. The right panel shows quantitative analysis of immunohistochemical staining measured by H‐score. **p < 0.01, ***p < 0.001 versus Sham. *n* = 3. (C) TUNEL staining was used to detect cell apoptosis on brain sections of mice subjected to dMCAO for different times. Cell nuclei were stained with DAPI. Scale bars = 100 μm. The right panel shows quantitative analysis of fluorescence intensity. *p < 0.05, ***p < 0.001 versus Sham. *n* = 3. (D) Double immunofluorescence staining detected the co‐localization of Bax with NeuN (neuronal marker) or Iba1 (marker of microglia/macrophage) on brain sections of mice subjected to dMCAO for 3 days. Scale bars = 100 μm. The right panels show immunofluorescence analysis of the co‐localization of Bax with NeuN or Iba1. (E) Cellular lysates of Neuro‐2a cells treated with OGD for different times were prepared and analyzed by Western blot for Bax, Bcl‐2, and Caspase‐3. The representative blots are shown on the left, whereas band intensities that were measured and normalized to β‐Actin are shown on the right. Data are represented as mean ± SD, **p < 0.01, ***p < 0.001 versus 0 h. *n* = 3. (F) Neuro‐2a cells were exposed to OGD for different times, and then immunohistochemical staining of Bax, Bcl‐2 and Caspase‐3 was conducted. Scale bars = 100 μm. (G) Neuro‐2a cells were treated as in (F). TUNEL staining was used to detect cellular apoptosis. Cell nuclei were stained with DAPI. Scale bars = 100 μm.

The above results have demonstrated that mitochondrial impairments elicited by ischemia/hypoxia trigger a cascade of events, including the opening of mPTP, lowering of the MMP, depleting of the cellular ATP, and increasing of the ROS generation, all of which ultimately lead to apoptotic cell death. We therefore evaluated temporal changes in the expression of caspase‐3 and Bcl‐2 family (Bax, Bcl‐2) genes that play crucial roles in mitochondrial dysfunction‐induced apoptosis in the ischemic brain tissues. As a result, the expression levels of pro‐apoptotic Bax and caspase‐3 were time‐dependently upregulated with the increased dMCAO time, but anti‐apoptotic Bcl2 expression exhibited an inverse alteration to Bax and caspase‐3 (Figure 2A). Moreover, a similar result was obtained by immunohistochemistry staining of these three proteins (Figure 2B). To provide additional evidence of ischemia/hypoxia‐induced apoptosis, we then performed TUNEL staining to analyze the apoptotic cell death occurring in ischemic brain tissues of dMCAO mice and confirmed that the number of apoptotic cells progressively increased with the ischemic duration, and peaked on 3 days post‐dMCAO (Figure 2C). These findings suggest that mitochondrial dysfunction contributes to cellular apoptosis in ischemic brain tissues.

To identify cell types that undergo apoptosis in response to ischemia/hypoxia, we performed immunofluorescence staining with anti‐NeuN (neuronal marker), anti‐Iba1 (microglial marker), and anti‐Bax, and observed that Bax was primarily co‐localized with the neuronal marker, suggesting that neuronal cells are the predominant cell type that underwent apoptosis in ischemic brain tissues (Figure 2D). Thus, the subsequent experiments focused on the effect of ischemia/hypoxia on the mitochondrial function of neuronal cells. Next, we utilized the cultured Neuro‐2a cells to validate the above findings in vivo. The results showed that the expression of Bax and caspase‐3 gradually increased with prolonged and continuous exposure of cells to OGD, accompanied by the progressive downregulation of Bcl‐2 expression level (Figure 2E). These observations were further confirmed by immunohistochemistry staining of Bax, Bcl2, and caspase‐3 (Figure 2F; Figure S1A). As expected, TUNEL staining revealed that OGD‐induced Neuro‐2a cell apoptosis was gradually enhanced with increasing OGD intervention time, reaching a peak at 4 h (Figure 2G; Figure S1B). Collectively, these findings suggest that mitochondrial dysfunction caused by ischemia/hypoxia leads to neuronal cell apoptosis in OGD‐treated Neuro‐2a cells as well as in the ischemic brain tissues of dMCAO mice.

To further establish a causal relationship between mitochondrial dysfunction and neuronal apoptosis, we shortened the time interval between two measurements of mitochondrial function and cell apoptosis from 2 to 1 h. As shown in Figure S1C–E, mitochondrial dysfunction (cellular ATP depletion, mPTP opening) occurred as early as 3 h post‐OGD, while cell apoptosis was only rarely detected at this time, and the apoptotic difference did not reach statistical significance until 4 h post‐OGD compared with the untreated control. These suggest that OGD‐induced mitochondrial damage precedes apoptotic cell death of neuronal cells, indicating a causal relationship between mitochondrial dysfunction and neuronal apoptosis.

### 3.3 NBP Attenuates Ischemia/Hypoxia‐Induced Mitochondrial Dysfunction in the Neuronal Cells and Brain Tissues

**FIGURE 3:**
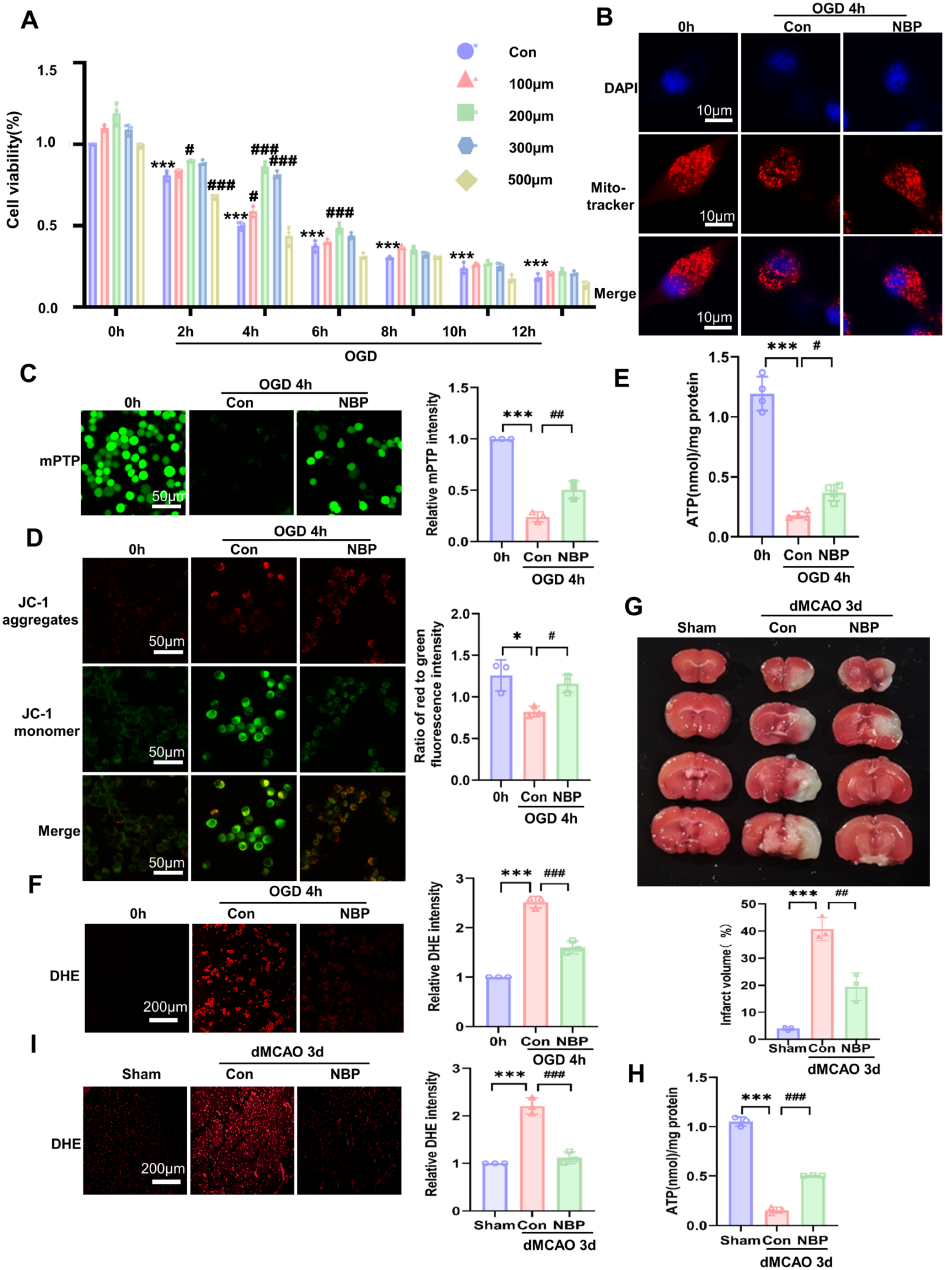
NBP attenuated ischemia/hypoxia‐induced mitochondrial dysfunction. (A) The viability of Neuro‐2a cells was measured by CCK‐8 assay after OGD‐exposed cells were treated with different concentrations of NBP and incubated for various times. ***p < 0.001 versus 0 h; ^#^ *p* < 0.05, ^###^ *p* < 0.001 versus OGD. *n* = 3. (B) Neuro‐2a cells were exposed to OGD and treated or not with NBP for 4 h, and mitochondria were stained with MitoTracker Red and observed under a confocal microscope. The nucleus was stained with DAPI. Scale bars = 10 μm. (C) Neuro‐2a cells were treated as in (B), and then mPTP opening was assessed by the quenching of calcein fluorescence with cobalt. The representative images are shown on the left. Scale bar = 50 μm. The right panel shows quantitative analysis of fluorescence intensity. ***p < 0.001 versus 0 h, ^##^ *p* < 0.01 versus Con. *n* = 3. (D) Neuro‐2a cells were treated as in (B), and MMP was detected by MMP Assay Kit with JC‐1. Relative ratio of red to green JC‐1 fluorescence intensity was measured. The representative fluorescence images are shown on the left. Scale bars = 50 μm. A quantitative analysis of fluorescence intensity is shown on the right. *p < 0.05 versus 0 h, ^#^ *p* < 0.05 versus Con. *n* = 3. (E) Neuro‐2a cells were treated as in (B), the ATP content was measured by using an ATP assay kit. Data are represented as mean ± SD, ***p < 0.001 versus 0 h, ^#^ *p* < 0.05 versus Con. *n* = 3. (F) Neuro‐2a cells were treated as in (B), and ROS levels were measured by dihydroethidium (DHE) staining. The representative fluorescence images are shown on the left. Scale bar = 200 μm. The right panel shows quantitative analysis of fluorescence intensity. ***p < 0.001 versus 0 h, ^###^ *p* < 0.001 versus Con. *n* = 3. (G) The representative TTC‐stained brain sections of Sham‐operated, dMCAO, and NPB‐treated dMCAO mice for 3 days are shown. The bar graph below shows the infarct volume (%) of each group of mice. Data are means ± SD, ***p < 0.001 versus Sham, ^##^ *p* < 0.001 versus Con. *n* = 3. (H) The ischemic cerebral tissues of dMCAO mice treated or not with NBP were used to determine ATP content by using an ATP detection kit. Data are represented as mean ± SD, ***p < 0.001 versus Sham, ^###^ *p* < 0.001 versus Con. *n* = 3. (I) The brain sections of dMCAO mice treated or not with NBP for 3 days were used to detect ROS by Dihydroethidium (DHE) staining. The representative fluorescence images are shown on the left. Scale bar = 200 μm. The right panel shows quantitative analysis of fluorescence intensity. ***p < 0.001 versus Sham, ^###^ *p* < 0.001 versus Con. *n* = 3.

Although existing studies have reported that NBP exerts neuroprotective effects by attenuating mitochondrial dysfunction [26, 27], detailed mechanisms of how NBP prevents the mitochondria of neuronal cells from ischemia/hypoxia injury remain to be fully investigated. To gain additional insights into how NBP protects the mitochondria against ischemia/hypoxia damage, we first performed CCK‐8 assays to evaluate the impact of OGD treatment on Neuro‐2a cell viability and NBP's protective effects against OGD‐induced cytotoxicity. Neuro‐2a cells were treated with different concentrations of NBP for various periods of time. As shown in Figure 3A, the viability of Neuro‐2a cells decreased with prolonged OGD treatment, with cell viability being reduced to approximately 50% at 4 h post‐OGD. Notably, the reduction in Neuro‐2a cell viability caused by OGD was significantly rescued by NBP treatment, with the most pronounced effects observed after cells were treated with 200 μM NBP for 4 h compared to other doses of NBP. Thus, we selected a dose of 200 μM of NBP to treat OGD‐exposed Neuro‐2a cells for 4 h in our subsequent in vitro experiments.

We next used Mito‐tracker Red staining to visualize the mitochondrial morphology in Neuro‐2a cells. The results showed that the mitochondria appeared as rod‐like structures in the cells treated with OGD for 0 h, but they became punctate structures (fissed mitochondria) after Neuro‐2a cells were subjected to OGD for 4 h. More importantly, OGD‐induced mitochondrial fission was largely abrogated by NBP treatment (Figure 3B). Subsequently, we examined whether mitochondrial impairments induced by OGD were improved following treatment with NBP. As shown in Figure 3C,D, OGD‐induced mPTP opening and MMP lowering were greatly reversed by NBP administration. Accordingly, NBP treatment partly counteracted the decrease in ATP content induced by OGD, while inhibiting ROS generation after OGD, as evidenced by DHE staining (Figure 3E,F). These data clearly suggest that NBP improves mitochondrial dysfunction by modulating mitochondrial morphology in OGD‐treated Neuro‐2a cells.

Based on the published literature [24, 25], a dose of 60 mg/kg/day of NBP was administered to dMCAO mice via oral gavage to validate the above findings. We first evaluated the protective effect of NBP on ischemic brain injury by assessing cerebral infarct volumes in dMCAO mice treated or not with NBP treatment, and results revealed that NBP treatment at 60 mg/kg significantly reduced the cerebral infarct size in dMCAO mice, indicating NBP's neuroprotective efficacy (Figure 3G). Additionally, a modified Longa scoring system was used to assess neurological function at 3 days after surgery. As shown in Figure S2A, the sham group mice showed a neurological score of zero, while the mice from the dMCAO and NBP groups had higher scores than the sham group. The NBP group showed significantly decreased neurological deficit scores compared with the dMCAO group. Further, we explored the impacts of NBP treatment on ATP content and ROS levels in ischemic brain tissues of dMCAO mice and demonstrated that, compared with the control group, ATP content was significantly increased, while ROS generation was attenuated, in NBP‐treated dMCAO mice (Figure 3H,I), consistent with the in vitro findings. Altogether, these results indicate that NBP can effectively mitigate ischemia/hypoxia‐induced mitochondrial dysfunction in the neuronal cells and brain tissues.

Simultaneously, we selected nicotinamide mononucleotide (NMN) and resveratrol to serve as two positive control drugs and to compare NBP's with NMN's or resveratrol's efficacy in improving mitochondrial function. Our results reveal that treating neuronal cells with 0.5 mM NMN exhibited similar effects as NBP, as evidenced by increased ATP content and decreased mPTP opening (Figure S2B,C), suggesting that mitochondrial protection elicited by NBP's inhibition of Drp1 acetylation is consistent with SIRT3‐mediated Drp1 deacetylation which is enhanced by replenishing NMN. While 100 μM resveratrol also counteracted OGD‐induced mitochondrial damage, its efficacy was lower than that of NBP (Figure S2B,C).

### 3.4 NBP Alleviates Ischemia/Hypoxia‐Induced Apoptosis by Improving the Impaired Mitochondrial Function

**FIGURE 4:**
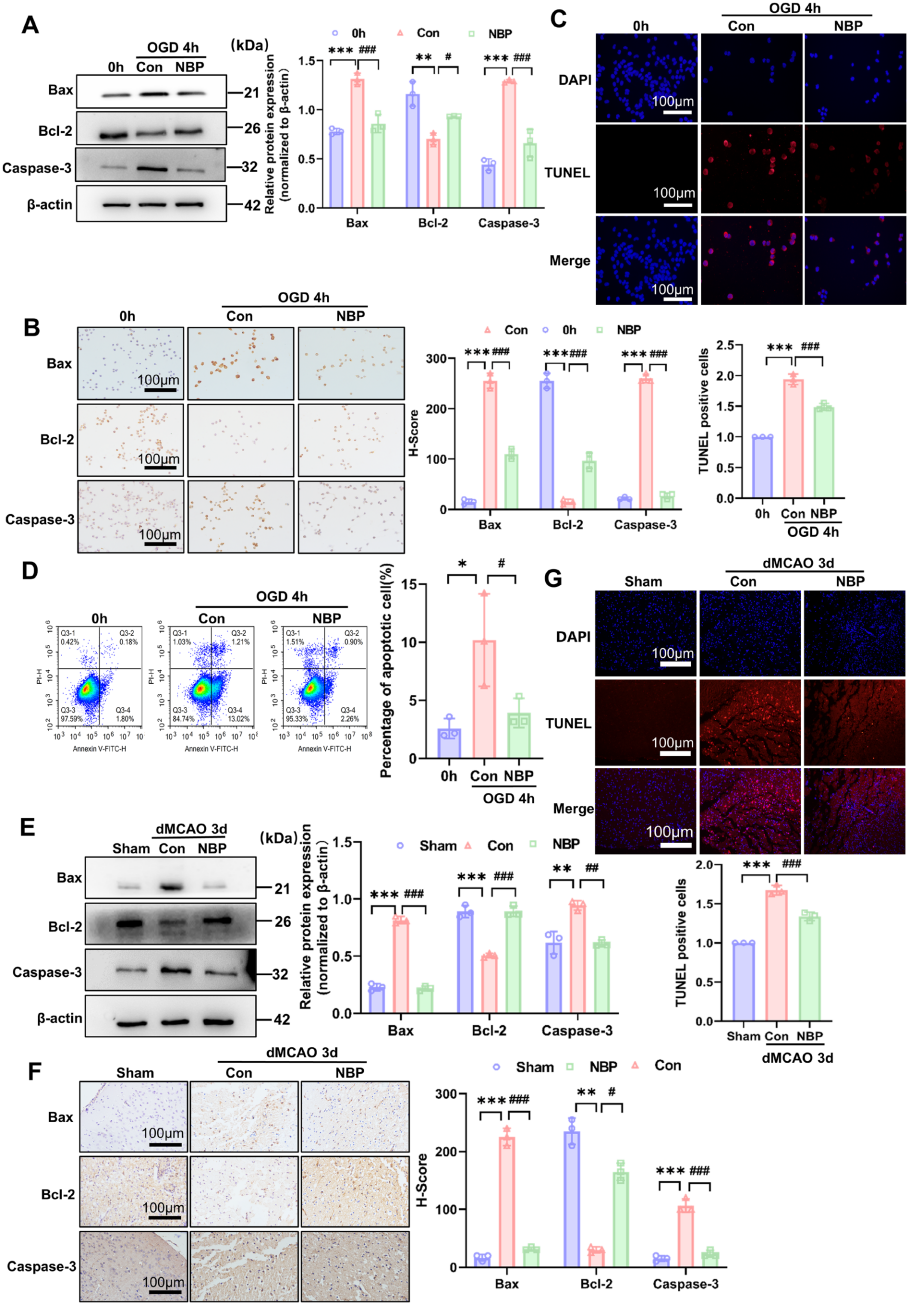
NBP alleviated ischemia/hypoxia‐induced cellular apoptosis in ischemic neuronal cells and brain tissues. (A) Neuro‐2a cells were exposed to OGD and treated or not with NBP for 4 h, and then cellular lysates were prepared and analyzed by Western blot for Bax, Bcl‐2, and Caspase‐3. A representative blot is shown on the left, whereas band intensities that were measured and normalized to β‐Actin are shown on the right. Data are represented as mean ± SD, **p < 0.01, and ***p < 0.001 versus 0 h; ^#^ *p* < 0.05, ^###^ *p* < 0.001 versus Con. *n* = 3. (B) Neuro‐2a cells were treated as in (A), and then immunohistochemical staining of Bax, Bcl‐2 and Caspase‐3 was conducted. Scale bars = 100 μm. The right panel shows quantitative analysis of immunohistochemical staining. ***p < 0.001 versus 0 h, ^###^ *p* < 0.001 versus Con. *n* = 3. (C) Neuro‐2a cells were treated as in (A), and TUNEL staining detected cellular apoptosis. Cell nuclei were stained with DAPI. Scale bars = 100 μm. The bottom panel shows quantitative analysis of TUNEL staining. ***p < 0.001 versus 0 h, ^###^ *p* < 0.001 versus Con. *n* = 3. (D) Neuro‐2a cells were treated as in (A), and flow cytometry analysis was applied to assess the rate of apoptosis. The data of flow cytometry were quantified and shown on the right. Data are represented as mean ± SD, *p < 0.05 versus 0 h, ^#^ *p* < 0.05 versus Con. *n* = 3. (E) The dMCAO mice were treated or not with NBP for 3 days, Protein extracts of brain tissues were prepared and analyzed by Western blot for Bax, Bcl‐2, and Caspase‐3. β‐Actin was included as a loading control. A representative blot is shown on the left, whereas band intensities that were measured and normalized to β‐Actin are shown on the right. Data are represented as mean ± SD, **p < 0.01, ***p < 0.001 versus Sham; ^##^ *p* < 0.01, and ^###^ *p* < 0.001 versus Con. *n* = 3. (F) The dMCAO mice were treated or not with NBP for 3 days, and immunohistochemical staining was performed to detect the expression of Bax, Bcl‐2 and Caspase‐3 on brain sections. Scale bars = 100 μm. The right panel shows quantitative analysis of immunohistochemical staining. **p < 0.01, and ***p < 0.001 versus Sham; ^#^ *p* < 0.05, and ^###^ *p* < 0.001 versus Con. *n* = 3. (G) The dMCAO mice were treated or not with NBP for 3 days, and TUNEL staining detected cellular apoptosis on mouse brain sections. Cell nuclei were stained with DAPI. Scale bars = 100 μm. The bottom panel shows quantitative analysis of TUNEL staining. ***p < 0.001 versus Sham, ^###^ *p* < 0.001 versus Con. *n* = 3.

We next wondered whether the restoration of impaired mitochondrial function by NBP administration can reduce cellular apoptosis in ischemic neuronal cells and brain tissues. We found that OGD‐induced upregulation of Bax and Caspase‐3 expression was abolished by NBP intervention in cultured Neuro‐2a cells (Figure 4A). Concomitantly, the decreased expression of the Bcl‐2 gene in OGD‐treated cells was recovered after NBP treatment (Figure 4A). Also, we performed immunohistochemical and immunofluorescence staining to evaluate the effects of NBP on the expression of Bax, Caspase‐3, and Bcl‐2 genes in OGD‐treated Neuro‐2a cells. As shown in Figure 4B and Figure S3A–C, the results obtained from the two staining methods were consistent with those obtained from Western blot analysis (Figure 4A), showing that NBP treatment decreased the upregulation of pro‐apoptotic Bax and Caspase‐3 induced by OGD, and negated the downregulation of anti‐apoptotic Bcl‐2 induced by OGD in the neuronal cells. We further employed TUNEL staining and flow cytometry to measure the impacts of NBP on OGD‐induced apoptosis in the neuronal cells. The results showed that the increased number of TUNEL‐positive cells caused by OGD was inversely changed by NBP (Figure 4C). Flow cytometric analysis yielded a similar result, showing that compared with OGD alone, NBP addition substantially blocked OGD‐induced apoptosis (Figure 4D). Further, we conducted animal experiments using the mouse dMCAO model. Western blot analysis revealed that NBP eliminated the upregulation of Bax and Caspase‐3 expression in ischemic brain tissues of dMCAO mice, while restoring the expression of Bcl‐2 (Figure 4E). Immunohistochemical staining of Bax, Bcl‐2 and Caspase‐3 also showed the same trend as that of Western blot analysis (Figure 4F). TUNEL staining further verified that the increased apoptosis in the ischemic brain tissues of dMCAO mice was largely alleviated by NBP treatment (Figure 4G). Overall, these results indicate that NBP alleviates ischemia/hypoxia‐induced apoptosis by improving the impaired mitochondrial function in neuronal cells and brain tissues.

### 3.5 NBP Reverses the Upregulation of Drp1 and GCN5L1 Expression by Ischemia/Hypoxia in Neuronal Cells and Brain Tissues

**FIGURE 5:**
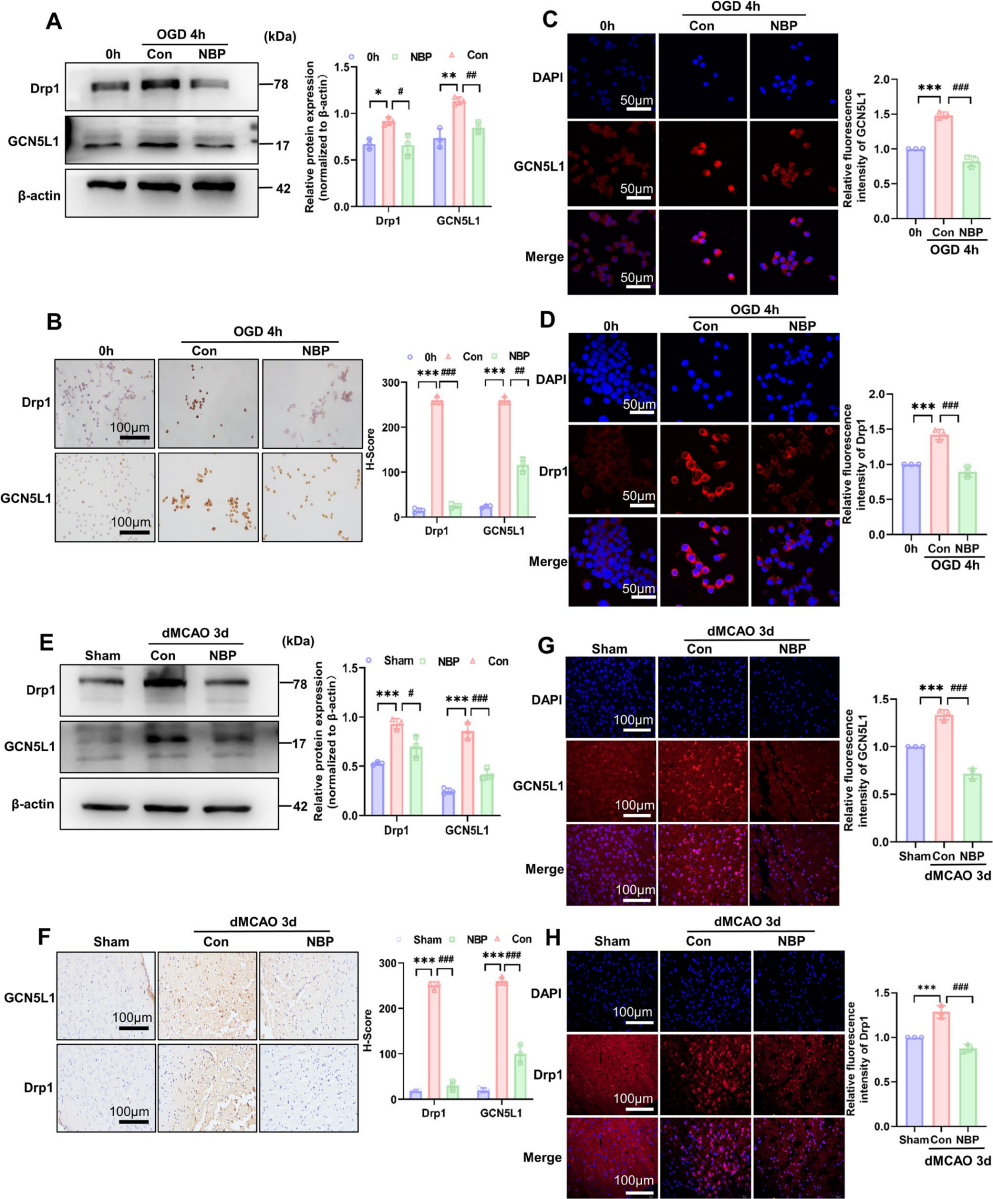
NBP reversed the upregulation of Drp1 and GCN5L1 expression by ischemia/hypoxia. (A) Neuro‐2a cells were exposed to OGD and treated or not with NBP for 4 h, and then cellular lysates were prepared and analyzed by Western blot for Drp1 and GCN5L1. The representative blots are shown on the left, whereas band intensities that were measured and normalized to β‐Actin are shown on the right. Data are represented as mean ± SD, *p < 0.05, **p < 0.01 versus 0 h; ^#^ *p* < 0.05, ^##^ *p* < 0.01 versus Con. *n* = 3. (B) Neuro‐2a cells were treated as in (A), and then immunohistochemical staining detected Drp1 and GCN5L1 expression. The representative images *are* shown on the left. Scale bars = 100 μm. The right panel shows quantitative analysis of immunohistochemical staining. ***p < 0.001 versus 0 h; ^##^ *p* < 0.01, ^###^ *p* < 0.001 versus Con. *n* = 3. (C, D) Neuro‐2a cells were treated as in (A), and then immunofluorescent staining detected the expression of GCN5L1 and Drp1. The nucleus was stained with DAPI. The representative fluorescence images are shown on the left. Scale bars = 50 μm. The right panels show quantitative analysis of their fluorescence intensity. ***p < 0.001 versus 0 h, ^###^ *p* < 0.001 versus Con. *n* = 3. (E) The dMCAO mice were treated or not with NBP for 3 days, and then protein extracts of brain tissues were prepared and analyzed by Western blot for Drp1 and GCN5L1. β‐Actin was included as a loading control. The representative blots are shown on the left, whereas band intensities that were measured and normalized to β‐Actin are shown on the right. Data are represented as mean ± SD, ***p < 0.001 versus Sham; ^#^ *p* < 0.05, and ^###^ *p* < 0.001 versus Con. *n* = 3. (F) The dMCAO mice were treated as in (E), and immunohistochemical staining detected the expression of GCN5L1 and Drp1 on brain sections. Scale bars = 100 μm. The right panel shows quantitative analysis of immunohistochemical staining. ***p < 0.001 versus Sham, ^###^ *p* < 0.001 versus Con. *n* = 3. (G, H) The dMCAO mice were treated as in (E), and then immunofluorescent staining detected the expression of GCN5L1 and Drp1. The nucleus was stained with DAPI. The representative fluorescence images are shown on the left. Scale bars = 100 μm. The right panels show quantitative analysis of their fluorescence intensity. ***p < 0.001 versus Sham, ^###^ *p* < 0.001 versus Con. *n* = 3.

Because our recent studies have demonstrated that dynamin‐related protein 1 (Drp1), whose activity is regulated by the mitochondrial acetyltransferase GCN5L1, exerted a key effect on mitochondrial injury under cerebral ischemic conditions, and that neuronal or cerebral ischemia/hypoxia resulted in excessive mitochondrial fission through upregulating the expression of Drp1 and GCN5L1, leading to mitochondrial dysfunction [14], we sought to determine whether the protective effects of NBP against mitochondrial damage and neuronal apoptosis depend on its regulation of the expression of Drp1 and GCN5L1. Thus, we investigated the effects of NBP on ischemia/hypoxia‐induced expression of Drp1 and GCN5L1. As shown in Figure 5A, NBP treatment completely abrogated the upregulation of the expression of Drp1 and GCN5L1 induced by OGD in Neuro‐2a cells. Immunohistochemical and immunofluorescence staining of Drp1 and GCN5L1 achieved similar results to Western blot analysis, further confirming that NBP suppressed ischemia/hypoxia‐induced expression of Drp1 and GCN5L1 in the neuronal cells (Figure 5B–D). In further experiments, we examined the expression of Drp1 and GCN5L1 in ischemic brain tissues of dMCAO mice following NBP treatment. The results showed that the upregulation of the two protein expressions caused by dMCAO surgery was substantially counteracted by the addition of NBP (Figure 5E). Consistent with the results of Western blot analysis, immunohistochemical and immunofluorescence staining also validated the decrease of Drp1 and GCN5L1 expression in brain tissues of dMCAO mice after NBP administration (Figure 5F–H). Collectively, these findings indicate that NBP treatment can abolish the upregulation of Drp1 and GCN5L1 expression induced by ischemia/hypoxia in neuronal cells and brain tissues.

### 3.6 NBP Decreases the Acetylation of Drp1 by GCN5L1 Through Prohibiting the Interaction Between Drp1 and GCN5L1

**FIGURE 6:**
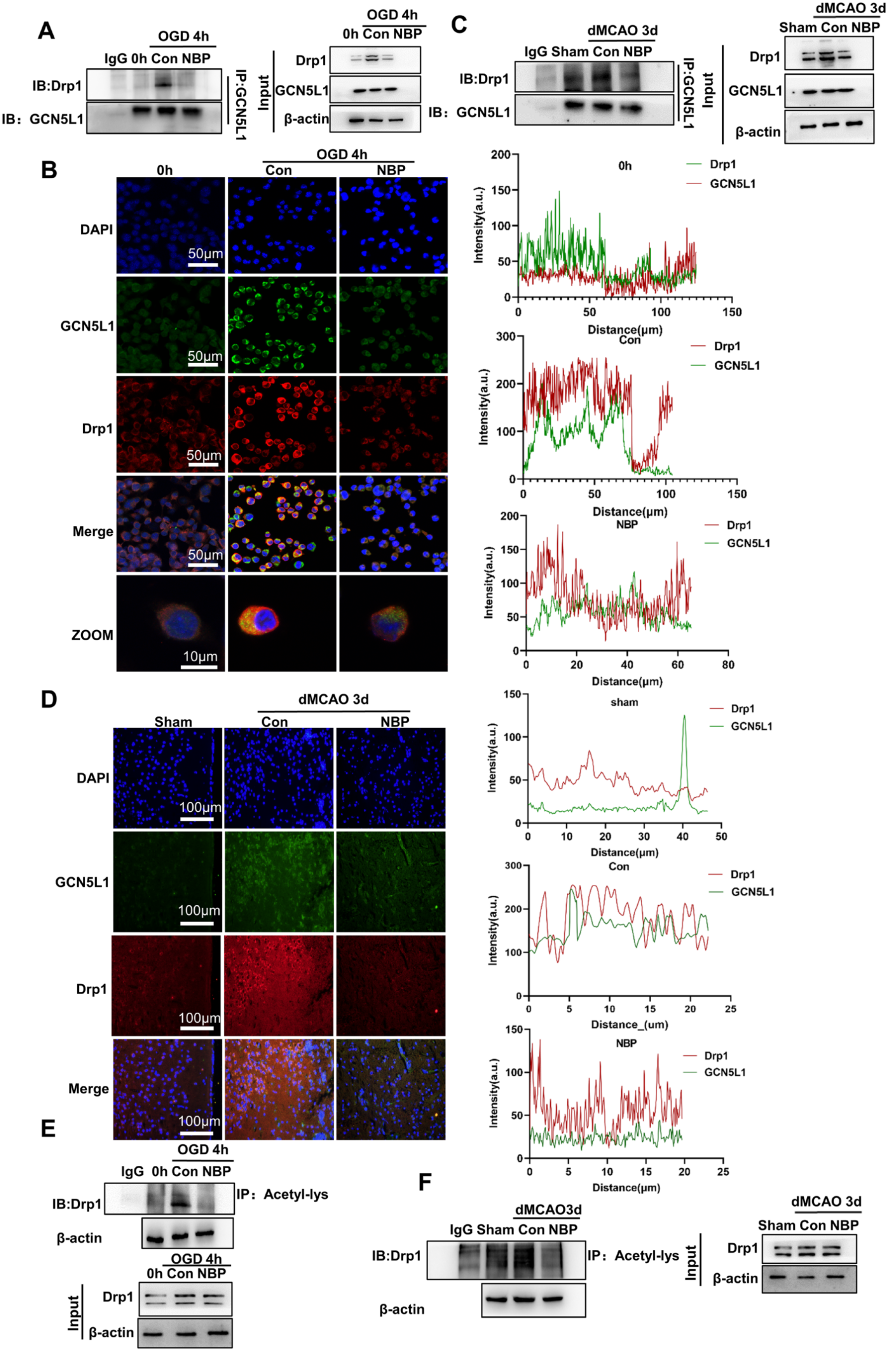
NBP decreased the acetylation of Drp1 by GCN5L1 by suppressing Drp1 interaction with GCN5L1. (A) Neuro‐2a cells were exposed to OGD and treated or not with NBP for 4 h, and then cellular lysates were prepared and immunoprecipitated with an anti‐GCN5L1 antibody. The immunoprecipitates were separated by SDS‐PAGE and immunoblotted with anti‐Drp1 antibody. IgG was used as a negative control for immunoprecipitation. (B) Neuro‐2a cells were treated as in (A), and then double immunofluorescence staining detected the expression and co‐localization of GCN5L1 (green) with Drp1 (red). The nucleus was stained with DAPI (blue). Yellow staining indicates co‐localization of GCN5L1 with Drp1. The representative fluorescence images are shown on the left. Scale bars = 50 μm and 10 μm, respectively. The right panels show immunofluorescence analysis of the colocalization of GCN5L1 with Drp1. (C) The dMCAO mice were treated or not with NBP for 3 days, protein extracts of brain tissues were prepared and immunoprecipitated with an anti‐GCN5L1 antibody. The immunoprecipitates were separated by SDS‐PAGE and immunoblotted with anti‐Drp1 antibody. IgG was used as a negative control for immunoprecipitation. (D) The dMCAO mice were treated as in (C), and double immunofluorescence staining detected the expression and co‐localization of GCN5L1 (green) with Drp1 (red) on brain sections. The nucleus was stained with DAPI (blue). Yellow staining indicates co‐localization of GCN5L1 with Drp1. The representative fluorescence images are shown on the left. Scale bars = 100 μm. The right panels show immunofluorescence analysis of the colocalization of GCN5L1 with Drp1. (E) Neuro‐2a cells were treated as in (A), and then cellular lysates were prepared and immunoprecipitated with anti‐acetyl‐lysine antibody. The immunoprecipitates were separated by SDS‐PAGE and immunoblotted with anti‐Drp1 antibody. IgG was used as a negative control for immunoprecipitation. (F) The dMCAO mice were treated as in (C), protein extracts of brain tissues were prepared and immunoprecipitated with anti‐acetyl‐lysine antibody. The immunoprecipitates were separated by SDS‐PAGE and immunoblotted with anti‐Drp1 antibody. IgG was used as a negative control for immunoprecipitation.

Considering that the above results have established that NBP repressed the expression of Drp1 and GCN5L1 induced by ischemia/hypoxia, and that our previous studies showed that GCN5L1 interacted with and acetylated Drp1 to increase Drp1 activity in ischemic neuronal cells and brain tissues [14], we reasoned that NBP might also regulate the acetylation status and activity of Drp1 via disrupting the interaction between GCN5L1 and Drp1. To verify this hypothesis, we conducted co‐immunoprecipitation (Co‐IP) experiments with anti‐GCN5L1, and the immunoprecipitates were immunoblotted with Drp1 or GCN5L1 antibodies. The results showed that in Neuro‐2a cells subjected to OGD, the association of GCN5L1 with Drp1 was significantly increased; however, their interaction was greatly weakened by NBP treatment (Figure 6A). Meanwhile, we exploited immunofluorescence co‐staining to assess the changes in colocalization between Drp1 and GCN5L1 in OGD‐exposed cells treated or not with NBP. As expected, the increased colocalization of Drp1 with GCN5L1 in OGD‐treated cells was markedly decreased by NBP treatment (Figure 6B), indicating that besides suppressing the Drp1 and GCN5L1 expression, NBP also inhibited the interaction of GCN5L1 with Drp1. These observations were also supported by experimental studies in dMCAO mice, showing that the interaction and colocalization of Drp1 with GCN5L1 were obviously induced in ischemic brain tissues of dMCAO mice, whereas NBP treatment completely reversed these changes, as evidenced by Western blot analysis and immunofluorescence staining (Figure 6C,D). We further investigated the influence of NBP on the acetylation of Drp1 induced by OGD and found that the acetylation level of Drp1 was markedly upregulated in OGD‐treated Neuro‐2a cells, with NBP treatment leading to an obvious decrease in Drp1 acetylation level (Figure 6E). Moreover, the in vivo results from dMCAO mice were similar to those obtained using OGD‐treated Neuro‐2a cells (Figure 6F). Taken together, these findings indicate that NBP reduces the acetylation of Drp1 by GCN5L1 through prohibiting the interaction between Drp1 and GCN5L1 in ischemic neuronal cells and brain tissues.

### 3.7 NBP Downregulates Drp1 Acetylation Level by Inhibiting the ERK‐Mediated Drp1 Phosphorylation

**FIGURE 7:**
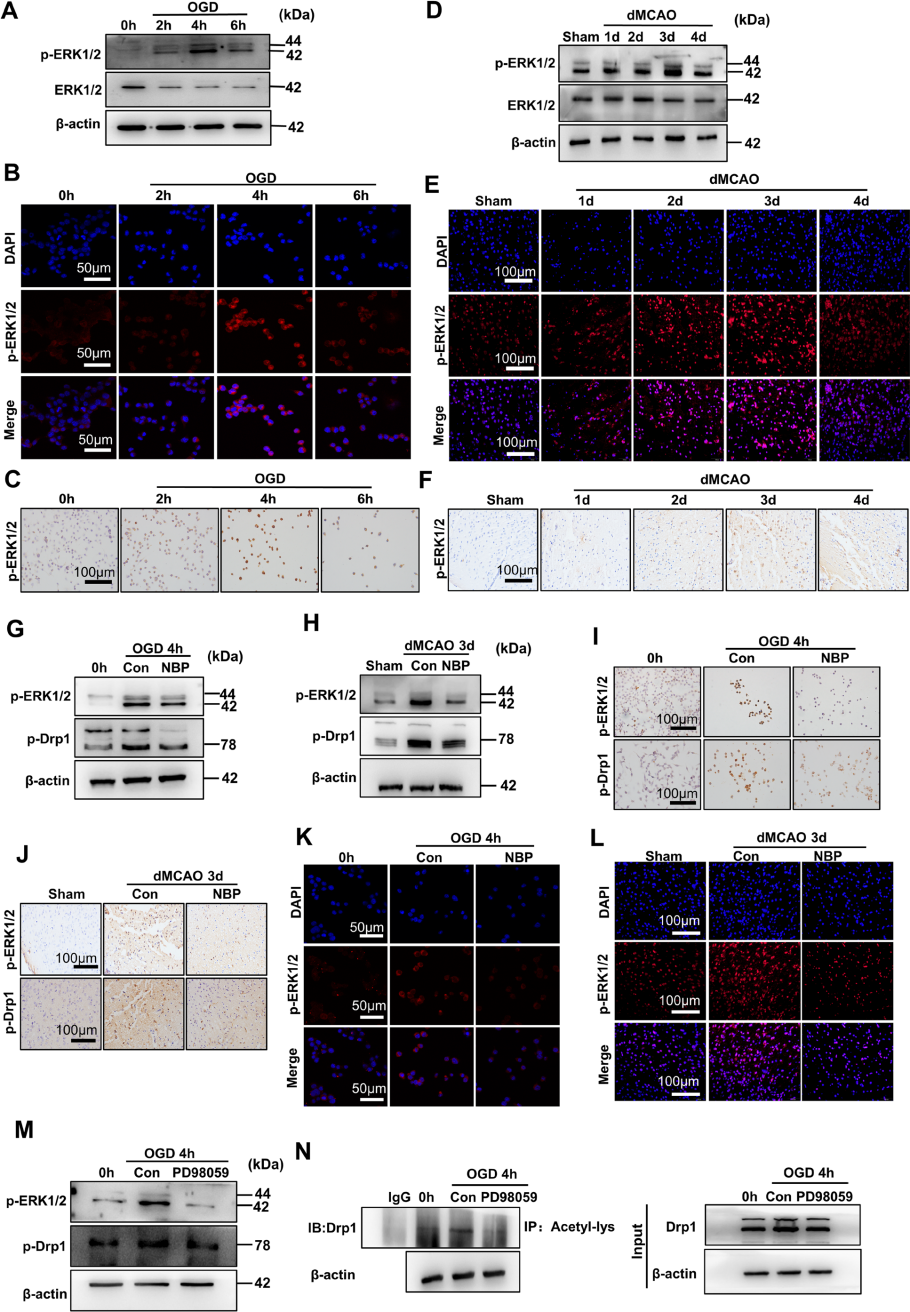
NBP downregulated Drp1 acetylation level by inhibiting ERK‐mediated Drp1 phosphorylation. (A) Neuro‐2a cells were exposed to OGD for 0, 2, 4, and 6 h, and then cellular lysates were prepared and analyzed by Western blot for p‐ERK1/2 and ERK1/2. β‐Actin was included as a loading control. (B) Neuro‐2a cells were treated as in (A), and immunofluorescent staining detected the expression of p‐ERK1/2. The nucleus was stained with DAPI. Scale bars = 50 μm. (C) Neuro‐2a cells were treated as in (A), and immunohistochemical staining detected p‐ERK1/2 expression. Scale bar = 100 μm. (D) Protein extracts of mouse brain subjected to dMCAO for 1, 2, 3, and 4 days were prepared and analyzed by Western blot for p‐ERK1/2 and ERK1/2. β‐Actin was included as a loading control. (E) Immunofluorescence staining detected p‐ERK1/2 expression on brain sections of mice subjected to dMCAO for different times. The nucleus was stained with DAPI. Scale bars = 100 μm. (F) Immunohistochemical staining detected the expression of p‐ERK1/2 on brain sections of mice subjected to dMCAO for different times. Scale bar = 100 μm. (G) Neuro‐2a cells were exposed to OGD and treated or not with NBP for 4 h, and then cellular lysates were prepared and analyzed by Western blot for p‐ERK1/2 and p‐Drp1. (H) The dMCAO mice were treated or not with NBP for 3 days, and then protein extracts of brain tissues were prepared and analyzed by Western blot for p‐ERK1/2 and p‐Drp1. (I) Neuro‐2a cells were treated as in (G), and immunohistochemical staining detected the expression of p‐ERK1/2 and p‐Drp1. Scale bars = 100 μm. (J) The dMCAO mice were treated as in (H), and immunohistochemical staining detected the expression of p‐ERK1/2 and p‐Drp1 on brain sections. Scale bars = 100 μm. (K) Neuro‐2a cells were treated as in (G), and immunofluorescent staining detected the expression of p‐ERK1/2. The nucleus was stained with DAPI. Scale bars = 50 μm. (L) The dMCAO mice were treated as in (H), and immunofluorescence staining detected the expression of p‐ERK1/2 on brain sections. The nucleus was stained with DAPI. Scale bars = 100 μm. (M) Neuro‐2a cells were exposed to OGD and treated or not with PD98059, and then cellular lysates were prepared and analyzed by Western blot for p‐ERK1/2 and p‐Drp1. (N) Neuro‐2a cells were treated as in (M), and then cellular lysates were prepared and immunoprecipitated with anti‐acetyl‐lysine antibody. The immunoprecipitates were separated by SDS‐PAGE and immunoblotted with anti‐Drp1 antibody. IgG was used as a negative control for immunoprecipitation.

Because protein phosphorylation is the most common post‐translational modification of proteins and frequently regulates specific protein–protein interactions, and because it has been reported that Drp1 could be phosphorylated by ERK1/2 signaling in cardiomyocytes [28], we next explored whether the ERK1/2‐mediated phosphorylation of Drp1 is responsible for cerebral hypoxia/ischemia‐induced GCN5L1 interaction with Drp1 and thus Drp1 acetylation. We first examined the impacts of the cellular hypoxia/ischemia on the phosphorylation of ERK1/2. As shown in Figure 7A and Figure S4A, the phosphorylation level of ERK1/2 significantly increased in Neuro‐2a cells subjected to OGD for different times, peaking at 4 h, and then decreased, without affecting the levels of total *ERK1/2*. Immunofluorescence and immunohistochemical staining of p‐*ERK1/2* yielded similar results to Western blot analysis, showing that ischemia/hypoxia caused by OGD in the neuronal cells contributed to the activation of ERK1/2 signaling (Figure 7B,C; Figure S4B,C). Subsequently, we used dMCAO mice to corroborate the findings obtained using the cultured Neuro‐2a cells and observed that p‐*ERK1/2* level was time‐dependently increased in response to dMCAO‐induced brain injury, reaching the highest level at 3 days post‐dMCAO (Figure 7D; Figure S4D). Moreover, the increased ERK1/2 phosphorylation in the ischemic brain tissues of dMCAO mice was further validated by immunofluorescence and immunohistochemical staining of p‐*ERK1/2* (Figure 7E,F; Figure S4E,F).

We then sought to evaluate whether the protective effects of NBP against ischemia/hypoxia injury in neuronal cells and brain tissues are related to its regulation of the phosphorylation of ERK1/2 and Drp1. To this end, we treated the OGD‐exposed cells and dMCAO mice with NBP and determined the effects of NBP administration on ERK1/2 and Drp1 phosphorylation. As shown in Figure 7G,H and Figure S4G,H, the upregulation of p‐ERK1/2 and p‐Drp1 levels by OGD in vitro and dMCAO in vivo was partly reversed by NBP treatment. To provide additional support for the Western blot results, we conducted immunohistochemical and immunofluorescence staining of the OGD‐exposed cells and ischemic brain tissues of dMCAO mice. As anticipated, NBP treatment drastically diminished the expression of p‐ERK1/2 and p‐Drp1 induced by OGD in vitro and dMCAO in vivo (Figure 7I–L; Figure S4I–L), indicating that the activation of ERK1/2 signaling is involved in the neuronal and brain injury caused by ischemia and hypoxia, whereas NBP might exert the protective effects on ischemic neuronal and brain tissues by interfering with ERK1/2 signaling. To further clarify whether ERK1/2 signaling mediates Drp1 phosphorylation caused by OGD, we incubated Neuro‐2a cells with the ERK inhibitor PD98059 for 1 h before exposure to OGD, and found that pharmacological inhibition of ERK1/2 blocked OGD‐induced Drp1 phosphorylation (Figure 7M), simultaneously accompanied by the decreased Drp1 acetylation (Figure 7N). These results clearly suggest that ERK1/2 signaling is the upstream signaling pathway leading to the phosphorylation of Drp1. Collectively, these data, together with the above‐mentioned findings, support the idea that NBP downregulates Drp1 acetylation levels through the inhibition of ERK1/2‐mediated Drp1 phosphorylation and Drp1 interaction with GCN5L1.

## 4 Discussion

Currently, standard treatments for acute ischemic stroke include intravenous thrombolysis with tissue plasminogen activator (tPA) and endovascular thrombectomy; however, these two therapeutic strategies are hampered by their limited therapeutic windows and associated risks such as hemorrhagic transformation [29, 30, 31]. As the powerhouse of the cell, mitochondria are particularly sensitive to changes caused by ischemia and hypoxia, making them the organelles most susceptible to damage [32, 33]. Therefore, the maintenance of mitochondrial function is crucial for neuron survival and neurological improvement, and there is a compelling need to develop neuroprotective agents capable of improving mitochondrial dysfunction.

NBP is a synthetic and important neuroprotective drug for the treatment of neurologic diseases. Its chemical formula is C_12_H_14_O_2_, and its molar mass is 190.24 g/mol. NBP is a fat‐soluble substance that can freely pass across the blood–brain barrier [22]. The role of NBP as a mitochondrial protector has been widely reported. For instance, Li and Zhu showed that NBP improves cerebral ischemia/reperfusion injury by regulating neuronal mitochondrial biogenesis via the AMPK/PGC‐1α signaling pathway [34]. Also, NBP attenuates mitochondrial impairments by reducing PARP1, PAR and p‐gH2AX levels [27]. Moreover, NBP attenuates cerebral ischemia/reperfusion injury in mice through AMPK‐mediated mitochondrial fusion [35]. Furthermore, NBP may improve cognitive impairment in chronic cerebral hypoperfusion (CCH) rats through the reduction of hippocampal neuron apoptosis by inhibiting mPTP opening and excessive mitophagy [36]. These studies fully demonstrate that NBP exerts protective roles against mitochondrial injury induced by ischemia/hypoxia through a multi‐target approach. Despite the critical roles of mitochondrial protein acetylation, particularly Drp1 acetylation, in regulating mitochondrial integrity and function, whether and how the protective effects of NBP on mitochondria are achieved by modulating Drp1 acetylation remains unexplored. The primary contribution of our research lies in providing strong evidence that NBP protects mitochondria against ischemia/hypoxia damage via suppressing GCN5L1‐mediated Drp1 acetylation. The main novelty of this work is as follows: (1) Ischemia/hypoxia upregulates Drp1 and GCN5L1 expression and induces ERK1/2 phosphorylation in Neuro‐2a cells and cerebral tissue. (2) NBP can reverse Drp1 and GCN5L1 upregulation by ischemia/hypoxia and suppress the phosphorylation of Drp1 by blocking the ERK1/2 signaling. (3) NBP attenuates the Drp1 and GCN5L1 interaction that depends on Drp1 phosphorylation, thereby decreasing Drp1 acetylation by GCN5L1 and excessive mitochondrial fission. Our work mechanistically extends the previous findings by defining the novel role of NBP in the regulation of Drp1 acetylation.

In the present study, when neuronal cells exposed to OGD were treated with NBP, the excessive mitochondrial fission caused by OGD was largely abrogated. Simultaneously, mitochondrial dysfunction resulted from ischemia/hypoxia was improved, showing an increase in MMP and ATP level, and closure of the mPTP as well as a decrease of ROS generation following NBP treatment. An improvement of mitochondrial dysfunction in dMCAO mice was also observed after NBP administration. These findings suggest that NBP can alleviate ischemia/hypoxia‐induced mitochondrial dysfunction via relieving the excessive mitochondrial fission. Moreover, using both the OGD in vitro ischemic model and dMCAO in vivo ischemic model, we showed that NBP treatment could significantly suppress the neuronal cell apoptosis elicited by ischemia/hypoxia.

Drp1 plays a critical role in the regulation of mitochondrial dynamics. Its activity is tightly regulated by post‐translational modifications (PTMs). Acetylation is a newly recognized post‐translational Drp1 modification that regulates its activity. For example, under oxidative stress, the acetylation of Drp1 at K711 facilitates the recruitment of cytoplasmic Drp1 to the mitochondria, further promoting its assembly into large oligomers, thereby inducing mitochondrial fission that affects mitochondrial morphology and function, ultimately leading to mitochondrial dysfunction and cell damage [37]. Mitochondrial deacetylase SIRT3 directly interacts with Drp1 and specifically deacetylates the K711 site, thus protecting against oxidative stress‐induced mitochondrial dysfunction by reducing Drp1 K711 acetylation in neuronal cells [37]. Moreover, another study reported that lipid overload creates a redox environment to activate Drp1 through facilitating the acetylation of Drp1 at K642, inducing mitochondrial fission and dysfunction in the hearts of high‐fat diet (HFD)‐fed mice as well as in cardiomyocytes [38]. Collectively, these studies clearly suggest that Drp1 acetylation at K711 in neuronal cells or at K642 in cardiomyocytes contributes to mitochondrial dysfunction and cell death elicited by ischemia/hypoxia or oxidative stress. Despite considerable progress elucidating the molecular regulation of Drp1 acetylation and pathophysiological significance, it remains unclear how Drp1 is acetylated in ischemic/hypoxic neuronal cells. Our study provides robust evidence that ERK signaling activated by ischemia/hypoxia enhances GCN5L1 association with Drp1 through inducing Drp1 phosphorylation; that is, Drp1 interaction with GCN5L1 depends on Drp1 phosphorylation, which promotes Drp1 acetylation by GCN5L1. NBP treatment protects mitochondria against ischemic/hypoxic damage by inhibiting the ERK‐mediated phosphorylation of Drp1 and thus impeding GCN5L1 interaction with Drp1, which leads to the downregulation of Drp1 acetylation level. Our present findings, together with the previous observations, support the notion that GCN5L1‐mediated Drp1 acetylation contributes to mitochondrial dysfunction in neurons, linking GCN5L1‐mediated Drp1 acetylation to ischemic stroke pathogenesis.

We are aware of the fact that our study has several limitations. (1) Although we have demonstrated that NBP can effectively alleviate mitochondrial dysfunction through inhibiting the ERK‐mediated phosphorylation of Drp1 and impeding GCN5L1 interaction with Drp1, the present study has not been able to demonstrate that the protective effects of NBP against mitochondrial injury rely also on its regulation of GCN5L1‐mediated Drp1 acetylation in ischemic stroke patients. (2) Given that NBP possesses the complex multi‐target neuroprotective effects, it remains unclear whether post‐translational modifications of other proteins mediated by ERK signaling also affect mitochondrial function. (3) This study was performed only using Neuro‐2a cells, a murine neuroblastoma cell line; thus, the molecular mechanism whereby NBP protects mitochondria against ischemic damage remains to be further validated using primary mouse neurons or human neurons such that it can enhance physiological relevance and improve the translational value of the findings. (4) Another limitation is that the specific acetylation sites of Drp1 acetylated by GCN5L1 have not been identified in this study, which will be addressed in future research.

## Author Contributions

Ya Wen conceptualized and designed the experiments. Haitao Zhang, Ning Zhang, Xiaotong Yang, Jiejie Zhang, Xiaoli Ge and Lei Wang performed all the experiments. Shan Wang analyzed and interpreted the data. Haitao Zhang and Ya Wen wrote the manuscript.

## Funding

The work was supported by Hebei Natural Science Foundation (No. H2024206168), National Natural Science Foundation of China (No. 82101378) and Construction of Provincial Excellent Characteristic Disciplines of Hebei Medical University (No. 2022LCTD‐B18).

## Ethics Statement

The study was reviewed and approved by the Laboratory Animal Welfare and Ethics Committee of the Second Hospital of Hebei Medical University (approval No: 2024‐AE100).

## Consent

The authors have nothing to report.

## Conflicts of Interest

The authors declare no conflicts of interest.

## Supporting information


**Figure S1:** (A) Quantitative analysis of immunohistochemical staining for Bax, Bcl‐2 and Caspase‐3 in Figure 2F. *p < 0.05, and ***p < 0.001 versus 0 h. *n* = 3. (B) Quantitative analysis of TUNEL staining in Figure 2G. **p < 0.01, ***p < 0.001 versus 0 h. *n* = 3. (C) Neuro‐2a cells were exposed to OGD for different times, and the ATP content was determined by using an ATP assay kit. Data are represented as mean ± SD, ***p < 0.001 versus 0 h. *n* = 3 for each group. (D) Neuro‐2a cells were exposed to OGD for different times, and mitochondrial permeability transition pore (mPTP) opening was assessed by the quenching of calcein fluorescence with cobalt. The representative fluorescence images are shown on the left. Scale bar = 50 μm. The right panel shows quantitative analysis of fluorescence intensity. ***p < 0.001 versus 0 h. *n* = 3. (E) TUNEL staining was used to detect cellular apoptosis. Cell nuclei were stained with DAPI. Scale bars = 100 μm. The right panel shows quantitative analysis of TUNEL staining. ***p < 0.001 versus 0 h. *n* = 3.
**Figure S2:** (A) The dMCAO mice were treated or not with NBP for 3 days, then Longa neurological scores were used to assess neurological function. Data are represented as mean ± SD, ***p < 0.001 versus Sham, ^###^ *p* < 0.001 versus Con. *n* = 3. (B) Neuro‐2a cells were exposed to OGD and treated or not with NBP, NMN, or resveratrol for 4 h, and then mitochondrial permeability transition pore (mPTP) opening was assessed by the quenching of calcein fluorescence with cobalt. Scale bar = 50 μm. The quantitative analysis of fluorescence intensity is shown below. ***p < 0.001 versus 0 h, ^##^ *p* < 0.01, ^###^ *p* < 0.001 versus Con. *n* = 3. (C) Neuro‐2a cells were exposed to OGD and treated or not with NBP, NMN, or resveratrol for 4 h, and then the ATP content was measured by using an ATP assay kit. Data are represented as mean ± SD, ***p < 0.001 versus 0 h, ^#^ *p* < 0.05, ^###^ *p* < 0.001 versus Con. *n* = 3.
**Figure S3:** (A–C) Neuro‐2a cells were exposed to OGD and treated or not with NBP for 4 h, and then immunofluorescent staining detected the expression of Bax, Bcl‐2 and Caspase‐3. The nucleus was stained with DAPI. The representative fluorescence images are shown on the left. Scale bars = 50 μm. The right panels show quantitative analysis of their fluorescence intensity. **p < 0.01, and ***p < 0.001 versus 0 h; ^#^ *p* < 0.05, ^##^ *p* < 0.01, and ^###^ *p* < 0.001 versus Con. *n* = 3.
**Figure S4:** (A) Quantitative analysis of the band intensity of p‐ERK1/2 and ERK1/2 in Figure 7A, using the Image J software, which was normalized to the band intensity of β‐actin. Data are represented as mean ± SD, *p < 0.05, **p < 0.01, and ***p < 0.001 versus 0 h. *n* = 3. (B) Quantitative analysis of the fluorescence intensity of p‐ERK1/2 in Figure 7B. Data are represented as mean ± SD, ***p < 0.001 versus 0 h. *n* = 3. (C) Quantitative analysis of the immunohistochemical staining of p‐ERK1/2 in Figure 7C. Data are represented as mean ± SD, ***p < 0.001 versus 0 h. *n* = 3. (D) Quantitative analysis of the band intensity of p‐ERK1/2 and ERK1/2 in Figure 7D, which was normalized to the band intensity of β‐actin. Data are represented as mean ± SD, *p < 0.05, **p < 0.01, and ***p < 0.001 versus Sham. *n* = 3. (E) Quantitative analysis of the fluorescence intensity of p‐ERK1/2 in Figure 7E. Data are represented as mean ± SD, *p < 0.05, ***p < 0.001 versus Sham. *n* = 3. (F) Quantitative analysis of the immunohistochemical staining of p‐ERK1/2 in Figure 7F. Data are represented as mean ± SD, *p < 0.05, ***p < 0.001 versus Sham. *n* = 3. (G) Quantitative analysis of the band intensities of p‐ERK1/2 and p‐Drp1 in Figure 7G, which was normalized to the band intensities of β‐actin. Data are represented as mean ± SD, ***p < 0.001 versus 0 h, ^###^ *p* < 0.001 versus Con. *n* = 3. (H) Quantitative analysis of the band intensities of p‐ERK1/2 and p‐Drp1 in Figure 7H, which was normalized to the band intensities of β‐actin. Data are represented as mean ± SD, *p < 0.05, ***p < 0.001 versus Sham; ^#^ *p* < 0.05, ^###^ *p* < 0.001 versus Con. *n* = 3. (I) Quantitative analysis of the immunohistochemical staining of p‐ERK1/2 and p‐Drp1 in Figure 7I. Data are represented as mean ± SD, **p < 0.01, ***p < 0.001 versus 0 h; ^##^ *p* < 0.01, ^###^ *p* < 0.001 versus Con. *n* = 3. (J) Quantitative analysis of the immunohistochemical staining of p‐ERK1/2 and p‐Drp1 in Figure 7J. Data are represented as mean ± SD, ***p < 0.001 versus Sham, ^###^ *p* < 0.001 versus Con. *n* = 3. (K) Quantitative analysis of the fluorescence intensity of p‐ERK1/2 in Figure 7K. Data are represented as mean ± SD, **p < 0.01 versus 0 h, ^#^ *p* < 0.05 versus Con. *n* = 3. (L) Quantitative analysis of the fluorescence intensity of p‐ERK1/2 in Figure 7L. Data are represented as mean ± SD, ***p < 0.001 versus Sham, ^###^ *p* < 0.001 versus Con. *n* = 3.

## Data Availability

The datasets used and/or analyzed during the present study are available from the corresponding author on reasonable request.
