# Supplementary material for: DL‐3‐n‐Butylphthalide Protects Mitochondria Against Ischemia/Hypoxia Damage via Suppressing GCN5L1‐Mediated Drp1 Acetylation in Neurons and Mouse Brains
Source: CNS Neurosci Ther. 2025 Nov 30;31(12):e70682. doi: 10.1002/cns.70682 (PMC12665616; doi:10.1002/cns.70682)
Supplement: Supplementary file 1 — Figure S1: (A) Quantitative analysis of immunohistochemical staining for Bax, Bcl‐2 and Caspase‐3 in Figure 2F. *p < 0.05, and ***p < 0.001 versus 0 h. n = 3. (B) Quantitative analysis of TUNEL staining in Figure 2G. **p < 0.01, ***p < 0.001 versus 0 h. n = 3. (C) Neuro‐2a cells were exposed to OGD for different times, and the ATP content was determined by using an ATP assay kit. Data are represented as mean ± SD, ***p < 0.001 versus 0 h. n = 3 for each group. (D) Neuro‐2a cells were exposed to OGD for different times, and mitochondrial permeability transition pore (mPTP) opening was assessed by the quenching of calcein fluorescence with cobalt. The representative fluorescence images are shown on the left. Scale bar = 50 μm. The right panel shows quantitative analysis of fluorescence intensity. ***p < 0.001 versus 0 h. n = 3. (E) TUNEL staining was used to detect cellular apoptosis. Cell nuclei were stained with DAPI. Scale bars = 100 μm. The right panel shows quantitative analysis of TUNEL staining. ***p < 0.001 versus 0 h. n = 3. Figure S2: (A) The dMCAO mice were treated or not with NBP for 3 days, then Longa neurological scores were used to assess neurological function. Data are represented as mean ± SD, ***p < 0.001 versus Sham, ### p < 0.001 versus Con. n = 3. (B) Neuro‐2a cells were exposed to OGD and treated or not with NBP, NMN, or resveratrol for 4 h, and then mitochondrial permeability transition pore (mPTP) opening was assessed by the quenching of calcein fluorescence with cobalt. Scale bar = 50 μm. The quantitative analysis of fluorescence intensity is shown below. ***p < 0.001 versus 0 h, ## p < 0.01, ### p < 0.001 versus Con. n = 3. (C) Neuro‐2a cells were exposed to OGD and treated or not with NBP, NMN, or resveratrol for 4 h, and then the ATP content was measured by using an ATP assay kit. Data are represented as mean ± SD, ***p < 0.001 versus 0 h, # p < 0.05, ### p < 0.001 versus Con. n = 3. Figure S3: (A–C) Neuro‐2a cells were exposed to OGD and t [file CNS-31-e70682-s001.zip › cns70682-sup-0001-FigureS1-S4@Supplementary Figure Legends.docx]

**Supplementary FIGURE S1:** (A) Quantitative analysis of immunohistochemical staining for Bax, Bcl-2 and Caspase-3 in Figure 2F. **P* < 0.05, and ****P* < 0.001 vs. 0 h. n =3. (B) Quantitative analysis of TUNEL staining in Figure 2G. ***P* < 0.01, ****P* < 0.001 vs. 0 h. n =3. (C) Neuro-2a cells were exposed to OGD for different times, and the ATP content was determined by using an ATP assay kit. Data are represented as mean ± SD, ****P*<0.001 vs. 0 h. n =3 for each group. (D) Neuro-2a cells were exposed to OGD for different times, and mitochondrial permeability transition pore (mPTP) opening was assessed by the quenching of calcein fluorescence with cobalt. The representative fluorescence images are shown on the left. Scale bar = 50 μm. The right panel shows quantitative analysis of fluorescence intensity. ****P* < 0.001 vs. 0 h. n =3. (E) TUNEL staining was used to detect cellular apoptosis. Cell nuclei were stained with DAPI. Scale bars = 100 μm. The right panel shows quantitative analysis of TUNEL staining. ****P*<0.001 vs. 0 h. n =3.

**Supplementary FIGURE S2:** (A) The dMCAO mice were treated or not with NBP for 3 days, then Longa neurological scores were used to assess neurological function. Data are represented as mean±SD, ***P < 0.001 vs. Sham, ^###^*P<*0.001 vs. Con. n =3. (B) Neuro-2a cells were exposed to OGD and treated or not with NBP, NMN, or resveratrol for 4 h, and then mitochondrial permeability transition pore (mPTP) opening was assessed by the quenching of calcein fluorescence with cobalt. Scale bar = 50 μm. The quantitative analysis of fluorescence intensity is shown below. ****P* < 0.001 vs. 0 h, ^##^*P<*0.01, ^###^*P<*0.001 vs. Con. n =3. (C) Neuro-2a cells were exposed to OGD and treated or not with NBP, NMN, or resveratrol for 4 h, and then the ATP content was measured by using an ATP assay kit. Data are represented as mean±SD, ****P*<0.001 vs. 0 h, ^#^*P<*0.05, ^###^*P<*0.001 vs. Con. n =3.

**Supplementary FIGURE S3:** (A-C) Neuro-2a cells were exposed to OGD and treated or not with NBP for 4 h, and then immunofluorescent staining detected the expression of Bax, Bcl-2 and Caspase-3. The nucleus was stained with DAPI. The representative fluorescence images are shown on the left. Scale bars = 50 μm. The right panels show quantitative analysis of their fluorescence intensity. **P < 0.01, and ***P < 0.001 vs. 0 h; ^#^*P<*0.05, ^##^*P<*0.01, and ^###^*P<*0.001 vs. Con. n =3.

**Supplementary FIGURE S4:** (A) Quantitative analysis of the band intensity of p-ERK1/2 and ERK1/2 in Figure 7A, using the Image J software, which was normalized to the band intensity of β-actin. Data are represented as mean±SD, **P*<0.05, ***P*<0.01, and ****P*<0.001 vs. 0 h. n=3. (B) Quantitative analysis of the fluorescence intensity of p-ERK1/2 in Figure 7B. Data are represented as mean±SD, ****P*<0.001 vs. 0 h. n=3. (C) Quantitative analysis of the immunohistochemical staining of p-ERK1/2 in Figure 7C. Data are represented as mean±SD, ****P*<0.001 vs. 0 h. n=3. (D) Quantitative analysis of the band intensity of p-ERK1/2 and ERK1/2 in Figure 7D, which was normalized to the band intensity of β-actin. Data are represented as mean±SD, **P*<0.05, ***P*<0.01, and ****P*<0.001 vs. Sham. n=3. (E) Quantitative analysis of the fluorescence intensity of p-ERK1/2 in Figure 7E. Data are represented as mean±SD, **P*<0.05, ****P*<0.001 vs. Sham. n=3. (F) Quantitative analysis of the immunohistochemical staining of p-ERK1/2 in Figure 7F. Data are represented as mean±SD, **P*<0.05, ****P*<0.001 vs. Sham. n=3. (G) Quantitative analysis of the band intensities of p-ERK1/2 and p-Drp1 in Figure 7G, which was normalized to the band intensities of β-actin. Data are represented as mean±SD, ****P*<0.001 vs. 0 h, ^###^*P<*0.001 vs. Con. n=3. (H) Quantitative analysis of the band intensities of p-ERK1/2 and p-Drp1 in Figure 7H, which was normalized to the band intensities of β-actin. Data are represented as mean±SD, **P*<0.05, ****P*<0.001 vs. Sham; ^#^*P<*0.05, ^###^*P<*0.001 vs. Con. n=3. (I) Quantitative analysis of the immunohistochemical staining of p-ERK1/2 and p-Drp1 in Figure 7I. Data are represented as mean±SD, ***P*<0.01, ****P*<0.001 vs. 0 h; ^##^*P<*0.01, ^###^*P<*0.001 vs. Con. n=3. (J) Quantitative analysis of the immunohistochemical staining of p-ERK1/2 and p-Drp1 in Figure 7J. Data are represented as mean±SD, ****P*<0.001 vs. Sham, ^###^*P<*0.001 vs. Con. n=3. (K) Quantitative analysis of the fluorescence intensity of p-ERK1/2 in Figure 7K. Data are represented as mean±SD, ***P*<0.01 vs. 0 h, ^#^*P<*0.05 vs. Con. n=3. (L) Quantitative analysis of the fluorescence intensity of p-ERK1/2 in Figure 7L. Data are represented as mean±SD, ****P*<0.001 vs. Sham, ^###^*P<*0.001 vs. Con. n=3.
