# Supplementary figures and images for: DL‐3‐n‐Butylphthalide Protects Mitochondria Against Ischemia/Hypoxia Damage via Suppressing GCN5L1‐Mediated Drp1 Acetylation in Neurons and Mouse Brains
Source: CNS Neurosci Ther. 2025 Nov 30;31(12):e70682. doi: 10.1002/cns.70682 (PMC12665616; doi:10.1002/cns.70682)

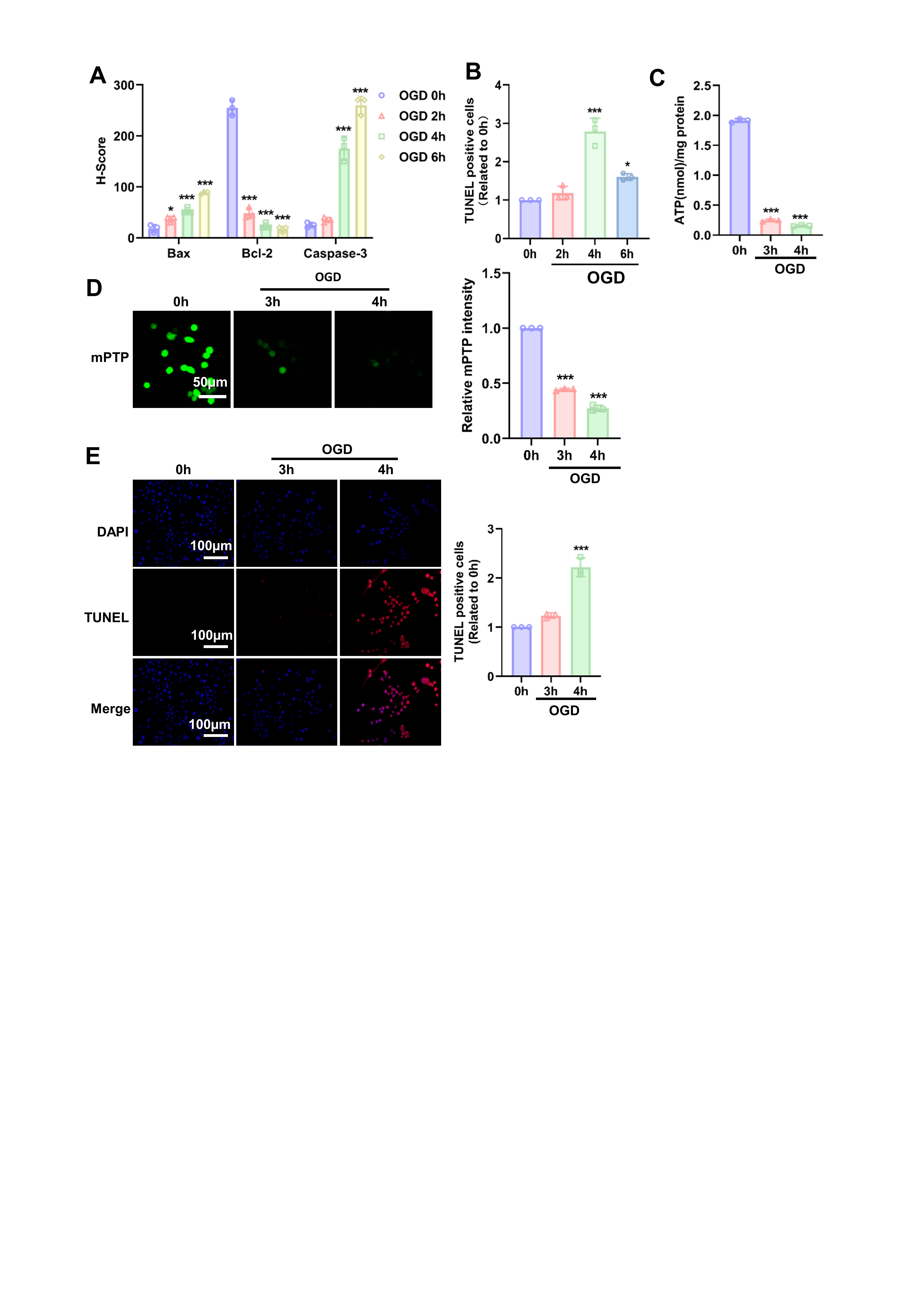

Supplement: Supplementary file 1 — Figure S1: (A) Quantitative analysis of immunohistochemical staining for Bax, Bcl‐2 and Caspase‐3 in Figure 2F. *p < 0.05, and ***p < 0.001 versus 0 h. n = 3. (B) Quantitative analysis of TUNEL staining in Figure 2G. **p < 0.01, ***p < 0.001 versus 0 h. n = 3. (C) Neuro‐2a cells were exposed to OGD for different times, and the ATP content was determined by using an ATP assay kit. Data are represented as mean ± SD, ***p < 0.001 versus 0 h. n = 3 for each group. (D) Neuro‐2a cells were exposed to OGD for different times, and mitochondrial permeability transition pore (mPTP) opening was assessed by the quenching of calcein fluorescence with cobalt. The representative fluorescence images are shown on the left. Scale bar = 50 μm. The right panel shows quantitative analysis of fluorescence intensity. ***p < 0.001 versus 0 h. n = 3. (E) TUNEL staining was used to detect cellular apoptosis. Cell nuclei were stained with DAPI. Scale bars = 100 μm. The right panel shows quantitative analysis of TUNEL staining. ***p < 0.001 versus 0 h. n = 3. Figure S2: (A) The dMCAO mice were treated or not with NBP for 3 days, then Longa neurological scores were used to assess neurological function. Data are represented as mean ± SD, ***p < 0.001 versus Sham, ### p < 0.001 versus Con. n = 3. (B) Neuro‐2a cells were exposed to OGD and treated or not with NBP, NMN, or resveratrol for 4 h, and then mitochondrial permeability transition pore (mPTP) opening was assessed by the quenching of calcein fluorescence with cobalt. Scale bar = 50 μm. The quantitative analysis of fluorescence intensity is shown below. ***p < 0.001 versus 0 h, ## p < 0.01, ### p < 0.001 versus Con. n = 3. (C) Neuro‐2a cells were exposed to OGD and treated or not with NBP, NMN, or resveratrol for 4 h, and then the ATP content was measured by using an ATP assay kit. Data are represented as mean ± SD, ***p < 0.001 versus 0 h, # p < 0.05, ### p < 0.001 versus Con. n = 3. Figure S3: (A–C) Neuro‐2a cells were exposed to OGD and t [file CNS-31-e70682-s001.zip › cns70682-sup-0002-FigureS1-S1@Supplementary Figure S1.tif]

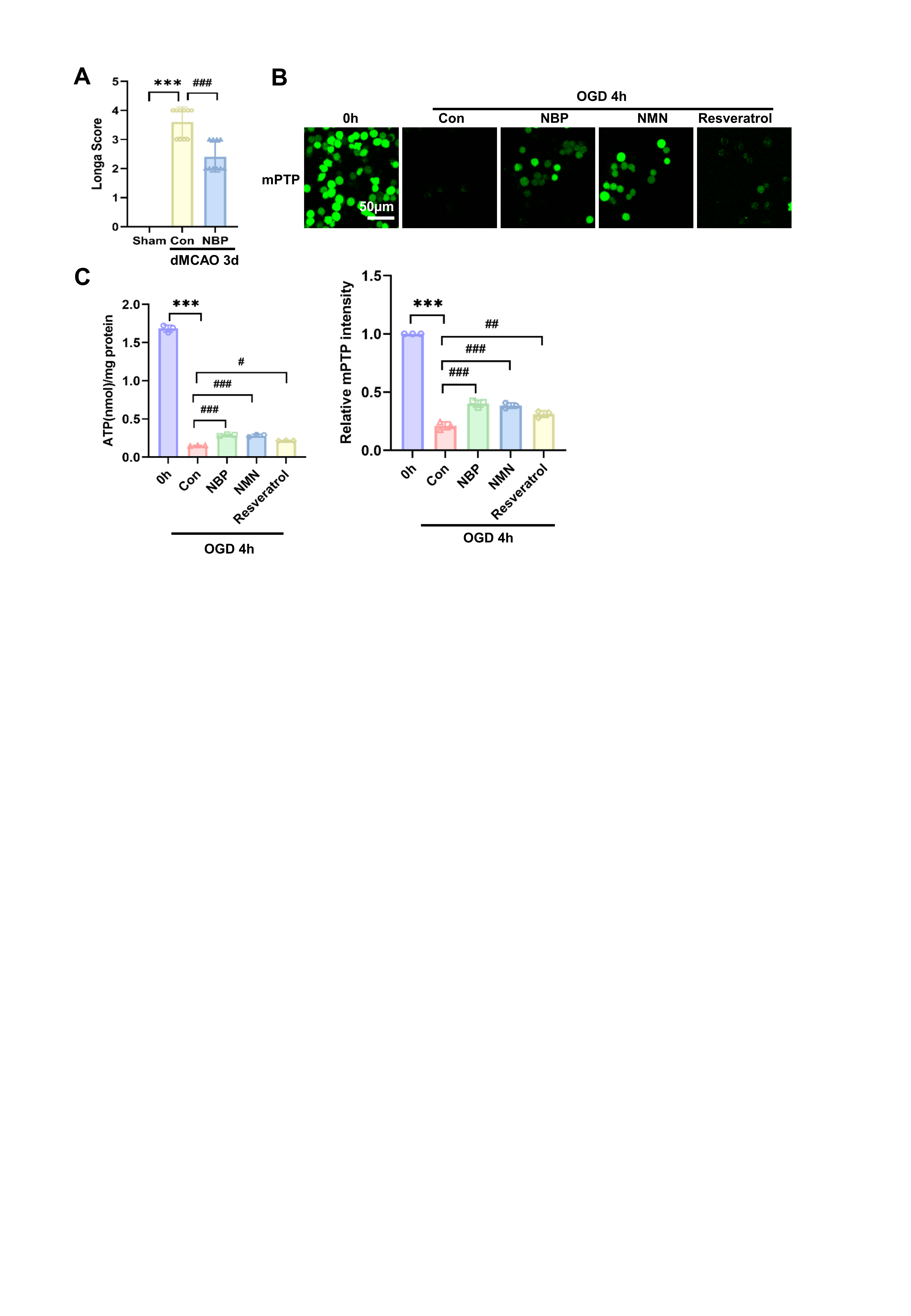

Supplement: Supplementary file 1 — Figure S1: (A) Quantitative analysis of immunohistochemical staining for Bax, Bcl‐2 and Caspase‐3 in Figure 2F. *p < 0.05, and ***p < 0.001 versus 0 h. n = 3. (B) Quantitative analysis of TUNEL staining in Figure 2G. **p < 0.01, ***p < 0.001 versus 0 h. n = 3. (C) Neuro‐2a cells were exposed to OGD for different times, and the ATP content was determined by using an ATP assay kit. Data are represented as mean ± SD, ***p < 0.001 versus 0 h. n = 3 for each group. (D) Neuro‐2a cells were exposed to OGD for different times, and mitochondrial permeability transition pore (mPTP) opening was assessed by the quenching of calcein fluorescence with cobalt. The representative fluorescence images are shown on the left. Scale bar = 50 μm. The right panel shows quantitative analysis of fluorescence intensity. ***p < 0.001 versus 0 h. n = 3. (E) TUNEL staining was used to detect cellular apoptosis. Cell nuclei were stained with DAPI. Scale bars = 100 μm. The right panel shows quantitative analysis of TUNEL staining. ***p < 0.001 versus 0 h. n = 3. Figure S2: (A) The dMCAO mice were treated or not with NBP for 3 days, then Longa neurological scores were used to assess neurological function. Data are represented as mean ± SD, ***p < 0.001 versus Sham, ### p < 0.001 versus Con. n = 3. (B) Neuro‐2a cells were exposed to OGD and treated or not with NBP, NMN, or resveratrol for 4 h, and then mitochondrial permeability transition pore (mPTP) opening was assessed by the quenching of calcein fluorescence with cobalt. Scale bar = 50 μm. The quantitative analysis of fluorescence intensity is shown below. ***p < 0.001 versus 0 h, ## p < 0.01, ### p < 0.001 versus Con. n = 3. (C) Neuro‐2a cells were exposed to OGD and treated or not with NBP, NMN, or resveratrol for 4 h, and then the ATP content was measured by using an ATP assay kit. Data are represented as mean ± SD, ***p < 0.001 versus 0 h, # p < 0.05, ### p < 0.001 versus Con. n = 3. Figure S3: (A–C) Neuro‐2a cells were exposed to OGD and t [file CNS-31-e70682-s001.zip › cns70682-sup-0003-FigureS2-S2@Supplementary Figure S2.tif]

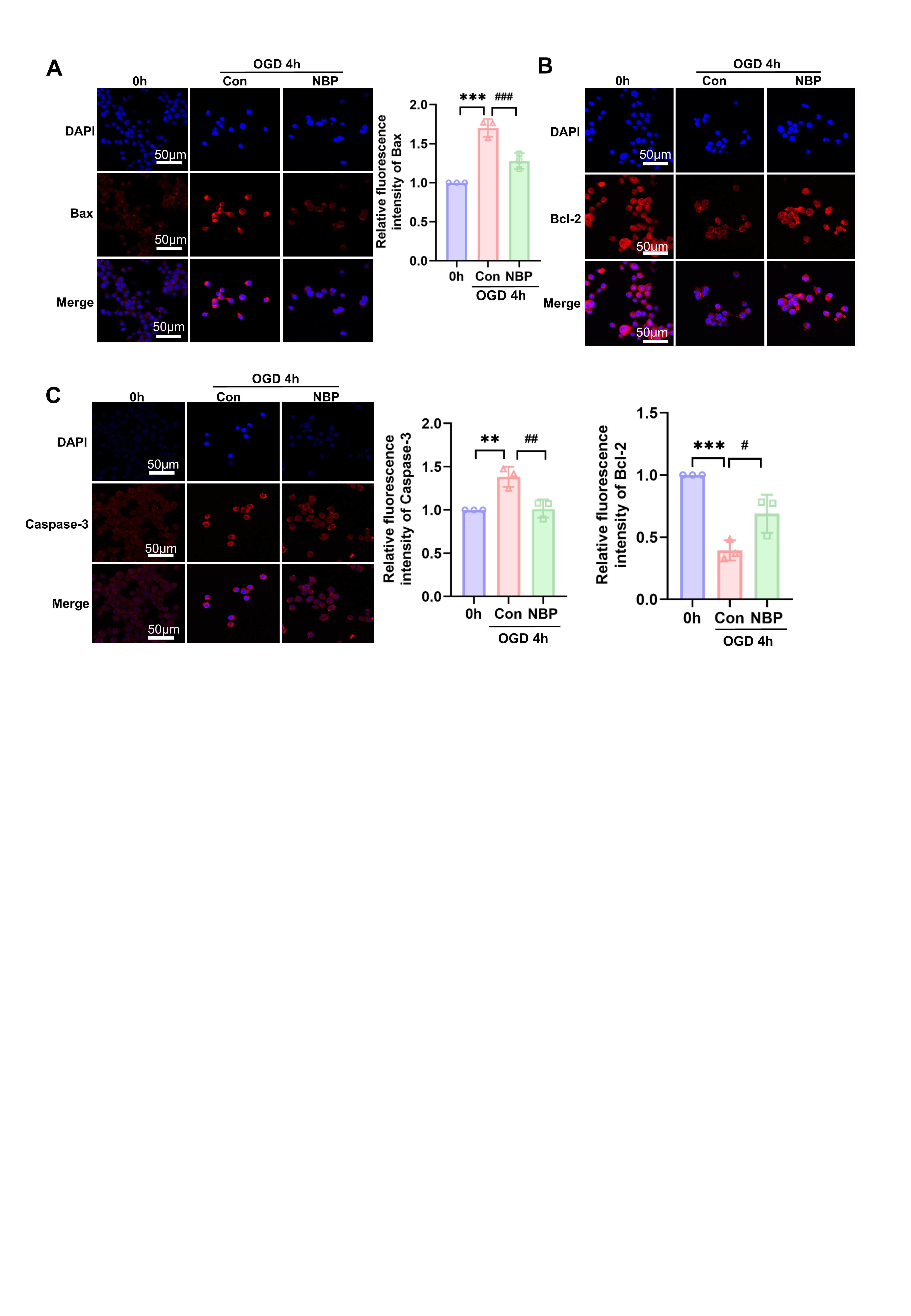

Supplement: Supplementary file 1 — Figure S1: (A) Quantitative analysis of immunohistochemical staining for Bax, Bcl‐2 and Caspase‐3 in Figure 2F. *p < 0.05, and ***p < 0.001 versus 0 h. n = 3. (B) Quantitative analysis of TUNEL staining in Figure 2G. **p < 0.01, ***p < 0.001 versus 0 h. n = 3. (C) Neuro‐2a cells were exposed to OGD for different times, and the ATP content was determined by using an ATP assay kit. Data are represented as mean ± SD, ***p < 0.001 versus 0 h. n = 3 for each group. (D) Neuro‐2a cells were exposed to OGD for different times, and mitochondrial permeability transition pore (mPTP) opening was assessed by the quenching of calcein fluorescence with cobalt. The representative fluorescence images are shown on the left. Scale bar = 50 μm. The right panel shows quantitative analysis of fluorescence intensity. ***p < 0.001 versus 0 h. n = 3. (E) TUNEL staining was used to detect cellular apoptosis. Cell nuclei were stained with DAPI. Scale bars = 100 μm. The right panel shows quantitative analysis of TUNEL staining. ***p < 0.001 versus 0 h. n = 3. Figure S2: (A) The dMCAO mice were treated or not with NBP for 3 days, then Longa neurological scores were used to assess neurological function. Data are represented as mean ± SD, ***p < 0.001 versus Sham, ### p < 0.001 versus Con. n = 3. (B) Neuro‐2a cells were exposed to OGD and treated or not with NBP, NMN, or resveratrol for 4 h, and then mitochondrial permeability transition pore (mPTP) opening was assessed by the quenching of calcein fluorescence with cobalt. Scale bar = 50 μm. The quantitative analysis of fluorescence intensity is shown below. ***p < 0.001 versus 0 h, ## p < 0.01, ### p < 0.001 versus Con. n = 3. (C) Neuro‐2a cells were exposed to OGD and treated or not with NBP, NMN, or resveratrol for 4 h, and then the ATP content was measured by using an ATP assay kit. Data are represented as mean ± SD, ***p < 0.001 versus 0 h, # p < 0.05, ### p < 0.001 versus Con. n = 3. Figure S3: (A–C) Neuro‐2a cells were exposed to OGD and t [file CNS-31-e70682-s001.zip › cns70682-sup-0004-FigureS3-S3@Supplementary Figure S3.tif]

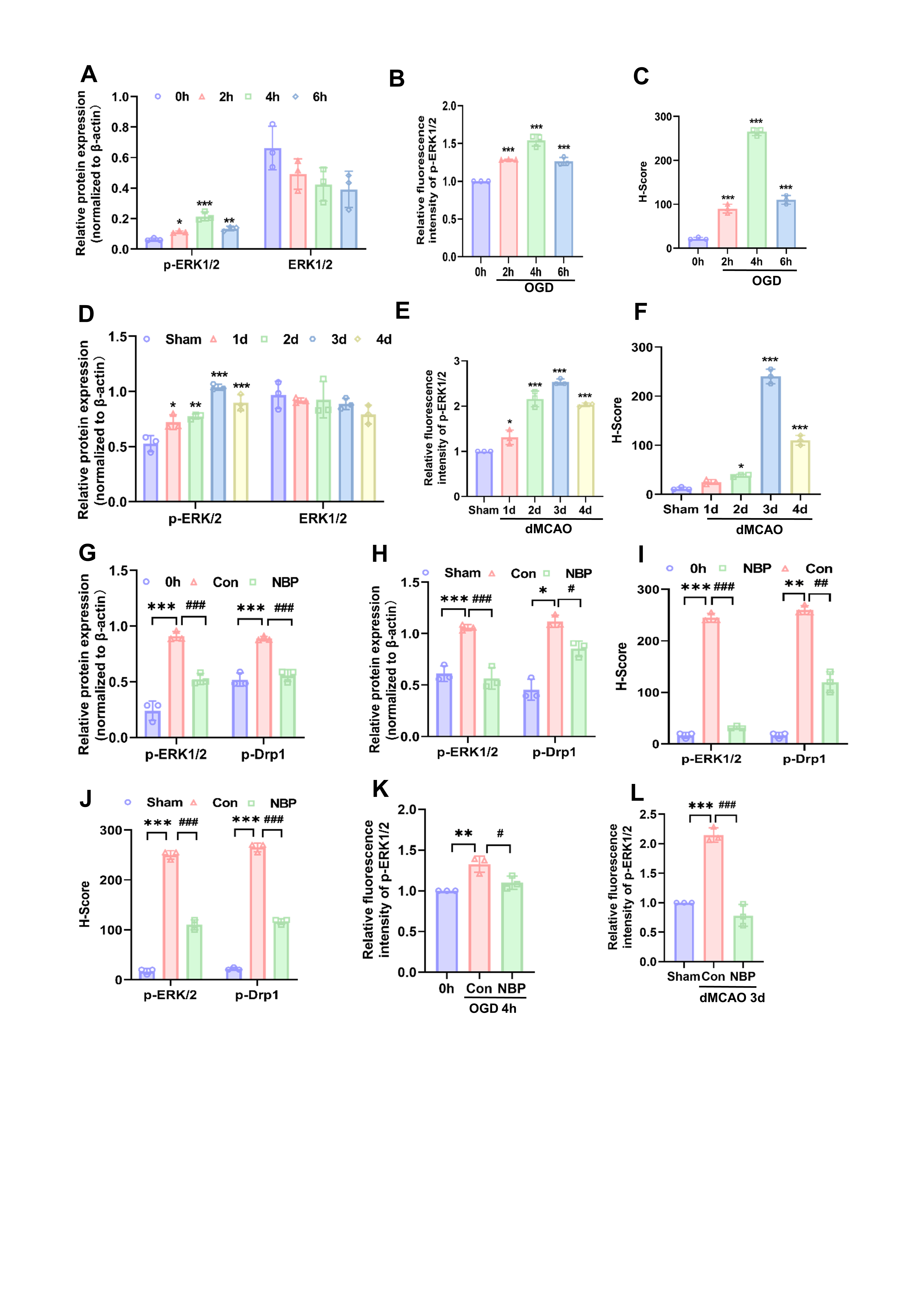

Supplement: Supplementary file 1 — Figure S1: (A) Quantitative analysis of immunohistochemical staining for Bax, Bcl‐2 and Caspase‐3 in Figure 2F. *p < 0.05, and ***p < 0.001 versus 0 h. n = 3. (B) Quantitative analysis of TUNEL staining in Figure 2G. **p < 0.01, ***p < 0.001 versus 0 h. n = 3. (C) Neuro‐2a cells were exposed to OGD for different times, and the ATP content was determined by using an ATP assay kit. Data are represented as mean ± SD, ***p < 0.001 versus 0 h. n = 3 for each group. (D) Neuro‐2a cells were exposed to OGD for different times, and mitochondrial permeability transition pore (mPTP) opening was assessed by the quenching of calcein fluorescence with cobalt. The representative fluorescence images are shown on the left. Scale bar = 50 μm. The right panel shows quantitative analysis of fluorescence intensity. ***p < 0.001 versus 0 h. n = 3. (E) TUNEL staining was used to detect cellular apoptosis. Cell nuclei were stained with DAPI. Scale bars = 100 μm. The right panel shows quantitative analysis of TUNEL staining. ***p < 0.001 versus 0 h. n = 3. Figure S2: (A) The dMCAO mice were treated or not with NBP for 3 days, then Longa neurological scores were used to assess neurological function. Data are represented as mean ± SD, ***p < 0.001 versus Sham, ### p < 0.001 versus Con. n = 3. (B) Neuro‐2a cells were exposed to OGD and treated or not with NBP, NMN, or resveratrol for 4 h, and then mitochondrial permeability transition pore (mPTP) opening was assessed by the quenching of calcein fluorescence with cobalt. Scale bar = 50 μm. The quantitative analysis of fluorescence intensity is shown below. ***p < 0.001 versus 0 h, ## p < 0.01, ### p < 0.001 versus Con. n = 3. (C) Neuro‐2a cells were exposed to OGD and treated or not with NBP, NMN, or resveratrol for 4 h, and then the ATP content was measured by using an ATP assay kit. Data are represented as mean ± SD, ***p < 0.001 versus 0 h, # p < 0.05, ### p < 0.001 versus Con. n = 3. Figure S3: (A–C) Neuro‐2a cells were exposed to OGD and t [file CNS-31-e70682-s001.zip › cns70682-sup-0005-FigureS4-S4@Supplementary Figure S4.tif]
